# Transcriptome-wide analysis reveals the molecular mechanisms of cannabinoid type II receptor agonists in cardiac injury induced by chronic psychological stress

**DOI:** 10.3389/fgene.2022.1095428

**Published:** 2023-01-10

**Authors:** Cheng Qin, Yujia Wang, Yang Zhang, Yan Zhu, Yabin Wang, Feng Cao

**Affiliations:** ^1^ Department of Cardiology, National Clinical Research Center for Geriatric Diseases and Second Medical Center of Chinese PLA General Hospital, Beijing, China; ^2^ Nankai University School of Medicine, Nankai University, Tianjin, China; ^3^ Beijing Key Laboratory of Research on Aging and Related Diseases, Beijing, China

**Keywords:** chronic psychological stress, endoplasmic reticulum stress, cardiac injuries, CB2R, ceRNA network, functional enrichment analysis

## Abstract

**Background:** Growing evidence has supported that chronic psychological stress would cause heart damage, However the mechanisms involved are not clear and effective interventions are insufficient. Cannabinoid type 2 receptor (CB2R) can be a potential treatment for cardiac injury. This study is aimed to investigate the protective mechanism of CB2R agonist against chronic psychological stress-induced cardiac injury.

**Methods:** A mouse chronic psychological stress model was constructed based on a chronic unpredictable stress pattern. Mice were performed a three-week psychological stress procedure, and cardiac tissues of them were collected for whole-transcriptome sequencing. Overlap analysis was performed on differentially expressed mRNAs (DE-mRNAs) and ER stress-related genes (ERSRGs), and bioinformatic methods were used to predict the ceRNA networks and conduct pathway analysis. The expressions of the DE-ERSRGs were validated by RT-qPCR.

**Results:** In the comparison of DE mRNA in Case group, Control group and Treatment group, three groups of ceRNA networks and ceRNA (circ) networks were constructed. The DE-mRNAs were mainly enriched in chromatid-relevant terms and Hematopoietic cell lineage pathway. Additionally, 13 DE-ERSRGs were obtained by the overlap analysis, which were utilized to establish a ceRNA network with 15 nodes and 14 edges and a ceRNA (circ) network with 23 nodes and 28 edges. Furthermore, four DE-ERSRGs (Cdkn1a, Atf3, Fkbp5, Gabarapl1) in the networks were key, which were mainly enriched in response to extracellular stimulus, response to nutrient levels, cellular response to external stimulus, and FoxO signaling pathway. Finally, the RT-qPCR results showed almost consistent expression patterns of 13 DE-ERSRGs between the transcriptome and tissue samples.

**Conclusion:** The findings of this study provide novel insights into the molecular mechanisms of chronic psychological stress-induced cardiac diseases and reveal novel targets for the cardioprotective effects of CB2R agonists.

## 1 Introduction

Psychological stress is a maladaptation in response to exposure to an environmental threat or severe external stimuli. Traumatic events, such as attacks, natural disasters, or military combat, can result in psychological stress, which is detrimental to the health of an individual (Glaser and Kiecolt-Glaser, 2005). A potential link might exist between psychological stress and cardiovascular diseases (CVD), a topic that has received increasing attention from scholars (Gold et al., 2020). Findings from multiple clinical studies have shown that psychological stress can increase the risk of adverse cardiovascular events, such as myocardial ischemia (Ebrahimi et al., 2021; Vaccarino et al., 2021; Shah et al., 2022), sudden death (Meisel et al., 1991; Leor et al., 1996), arrhythmia (Steinberg et al., 2004; Hourani et al., 2020), stroke (Jordan et al., 2013; Chen et al., 2015), and the aggravation of heart failure (Roy et al., 2015).

However, despite the epidemiological evidence, the pathophysiological mechanisms underlying these associations remain incomplete. Findings from recent studies suggest that autonomic dysfunction and inflammatory response promote plaque vulnerability, which is the cause of cardiovascular events induced by acute stress (Hinterdobler et al., 2021). However, the long-term effects of chronic stress on the cardiovascular system are not completely understood. Therefore, specific and effective interventions are urgently needed. Moreover, whether healthy individuals exposed to stress elicit similar pathophysiological responses as individuals with pre-existing cardiovascular diseases remains doubtful.

The endoplasmic reticulum (ER) is the primary site for protein synthesis, folding, and transport (Wang and Kaufman, 2016). The exposure of cells to oxidative stress, hypoxia, nutrient deprivation, and pathological infection will cause the accumulation of misfolded or unfolded proteins in the ER and initiate the unfolded protein response (UPR) (Ochoa et al., 2018). If stimuli persist, the UPR will lead to apoptosis and autophagy. ATF6, PERK, and IRE1 are the representative molecules of UPR signaling (Ron and Walter, 2007). Under usual conditions, these molecules bind to their partner molecule glucose-regulated protein 78 (GRP78). Under ER stress, unfolded or misfolded proteins recruit GRP78/BiP to dissociate from the complex composed of IRE1, ATF6, and PERK. This process marks the initiation of ER stress (Bertolotti et al., 2000; Frakes and Dillin, 2017). The expression of the abovementioned ER stress-related genes (ESRGs) was found to be related to the occurrence and development of various cardiovascular diseases, including myocardial ischemia (Bi et al., 2018; Zhang et al., 2019), ventricular remodeling (Blackwood et al., 2019), atherosclerosis (Tang et al., 2019), and vascular calcification (Fu et al., 2019). Likewise, the expression of ESRGs was found to be upregulated in the brain tissues of mice subjected to psychological stress (Gold et al., 2013; Mondal et al., 2015; Mao et al., 2019). Thus far, small molecule drugs targeting ESRGs have been developed, but they have not been adopted widely in clinical practice owing to their relative toxicity and lack of specificity (Atkins et al., 2013; Almanza et al., 2019).

Cannabinoid receptor type 2 (CB2R) is primarily localized in immune cells as well as in endothelial cells and cardiac tissues. It plays an important role in the regulation of the immune response and inflammation. CB2R agonists can exhibit a cardioprotective role in myocardial infarction, cardiomyopathy, arrhythmia, and other CVDs by reducing reactive oxygen species (ROS) production, inhibiting necroptosis and apoptosis, promoting efferocytosis, upregulating endothelial NO synthase, and reducing macrophage infiltration (Molica et al., 2012; Li et al., 2016; Yu et al., 2019; Liu X. et al., 2020). Owing to the anti-oxidative stress effect of CB2R agonists and the regulation of autophagy, CB2R agonists may affect the cellular process underlying ER stress.

Here, we aimed to elucidate the mechanism underlying cardiac injury induced by mental stress and the therapeutic targets of CB2R agonists. We constructed a mouse model induced by psychological stress (case group), stressed mice treated with CB2R agonists (treatment group), and normal mice (control group) and collected samples for whole transcriptome sequencing analysis to analyze the differences in gene expression profiles between the different groups.

## 2 Methods

### 2.1 Animal models

All mice were obtained from SPF Biotechnology and allowed 1 week of acclimation to the specific-pathogen-free animal facilities before the experiments were conducted. Six-week-old male mice were used in this study and were randomly divided into three groups: control group (three normal mice were maintained with free access to food and water on a 12 h light/dark cycle), case group (four mice with psychological stress-induced heart disease), and treatment group (four mice with psychological stress-induced heart disease treated with the CB2R agonist JWH133). JWH133 (ApexBio, B7941) was injected twice daily intraperitoneally at 10 mg/kg from the beginning of the stress induction procedure for 21 days. The stress induction process is shown inSupplementary Table S1. Behavioral tests were performed 24 h after stress induction.

### 2.2 Data sources

After stress induction, the hearts of the mice were removed, and the tissue was processed for whole-transcriptome sequencing analysis. After RNA extraction, purification, and library construction, these libraries were double-ended (paired-end, PE) sequenced by next-generation sequencing (NGS) using the Illumina HiSeq sequencing platform. The sequencing depth for the transcriptome-wide analysis was showed in Supplementary Figure S1.

In addition, 787 ERS-related genes with a relevance score of ≥7 were extracted from a report by Zhang et al. (2021). Since these genes were from a human gene set, the biomaRt package in R software was used to identify the homologous genes of mice. We identified 828 ERS-related genes in mice (Supplementary Table S2). GSE210252 was downloaded from the Gene Expression Omnibus (GEO) dataset (https://www.ncbi.nlm.nih.gov/), which contained four pairs samples from mice received restraint stress or no stress.

### 2.3 Behavioral test

#### 2.3.1 Open-field test

The open-field test can be used to observe voluntary movement and assess the severity of stress. The open-field system comprised an open-field box (40 × 40 × 40 cm^3^), an imaging system, and analysis software. During the experiment, the mice were placed in a specific position in the open-field, the camera system was used to monitor the activities of the animals in the open-field, and an analysis software was used to track the trajectory of the animals and collect data on their length of stay in the central area. The lesser time the mice remained in the central area, the more severe stress they experienced (Yan et al., 2022).

#### 2.3.2 Elevated plus maze (EPM)

EPM is an experimental tool used to evaluate the stress response of rodents. In this study, EPM was a cross-shaped platform composed of two open arms and two closed arms (arms 35 cm long × 5 cm wide) positioned approximately 55 cm above the ground. A monitoring camera for animal behavior experiments was installed above the EPM. The residence time in the open arm (open arm time, OT) was recorded using the Labmaze 3.0 animal behavior analysis software (Zhongshidichuang Sci. and Tech). For the EPM test, animals were placed in the center of the maze facing an open arm (Cho et al., 2022).

#### 2.3.3 Forced swimming test

The forced swimming test was conducted in a cylindrical water tank (50 cm in height and 10 cm in diameter). The water temperature was 23 ∼ 25 °C, and the water had a depth that was sufficient to position the tail of the animal at a certain distance from the bottom. The swimming time was 6 min, and the immobility time in the last 4 min of the swimming duration was recorded. Moreover, the standard of immobility was that the animal stopped struggling in the water and started floating, with only slight limb movement to keep the head afloat (Gu et al., 2021). A longer immobility time represented more severe stress.

#### 2.3.4 Tail suspension test

This experiment was conducted in a dark and quiet environment. The tail of the mouse was fixed and hung in a 25 cm × 25 cm × 30 cm box, and the head of the mouse was positioned approximately 5 cm from the bottom. Furthermore, the activity of the mouse was recorded within 6 min. The immobility time in the last 4 min was recorded. Small movements of the forelimbs rather than the hindlimbs were considered to represent immobility (Gu et al., 2021).

### 2.4 Differential analysis

The Limma package (version 3.44.3) was used to screen differentially expressed mRNAs (DE-mRNAs), miRNAs (DE-miRNAs), lncRNAs (DE-lncRNAs), and circRNAs (DE-circRNAs) in the three comparison groups (Control vs. Case; Case vs. Treatment; Control vs. Treatment). In detail, the count values obtained by comparing the mRNA and lncRNA data were converted to the fragments per kilobase per million mapped reads (FPKM) values. The count values used by miRNA and circRNA were converted to the counts per million mapped (CPM) value, and the selection standard was set at *p* < .05 and |Log_2_FC|>1. Moreover, the distribution of DE-mRNAs, DE-miRNAs, DE-circRNAs, and DE-lncRNAs in the three comparison groups was demonstrated at the chromosomal level using Circos in R package.

### 2.5 Construction of ceRNA networks

To predict target miRNAs that could potentially bind to the DE-mRNAs in the three comparison groups, mirwalk 3.0 was used with a threshold score >.95. Simultaneously, only the DE-miRNAs with opposite expression trends to DE-mRNAs were retained. After the target miRNA was obtained, starBase 2.0 was used to predict the miRNA-lncRNA pairs, and only the relationship pairs with opposite differential expression trends were retained. Thus, an mRNA-miRNA-lncRNA binding network was obtained from each comparison group, which was considered the ceRNA network.

Similarly, in each comparison group, mirwalk was used to predict the binding of DE-mRNA and DE-miRNA with a threshold score >.95, and only the pairs with contrasting trends of differential expression were retained. To predict the circRNAs that might be related to the miRNAs after obtaining miRNAs, the circRNA sequences were extracted from assembled gene files, miRNA sequences of mice were retrieved from miRbase, and the miRanda software was used to predict the binding with the selection criteria as binding score = 140, and only the opposite relationship pairs of differential expression trends were retained. Thus, an mRNA-miRNA-circRNA network was constructed for each group, known as the ceRNA (circ) network.

### 2.6 Screening and function annotation of differentially expressed ER stress-related genes (DE-ERSRGs)

Overlap analysis was performed on the DE-mRNAs from the Case vs. Control and Treatment vs. Case comparison groups and the 828 mouse ER stress-related genes. The intersected genes were considered to be DE-ERSRGs.

### 2.7 Construction of the DE-ERSRGs-based ceRNA network

Initially, mirwalk was used to predict the target miRNAs that can potentially bind to the DE-ERSRGs with a threshold score >.95. Simultaneously, only miRNAs in the pairs with contrasting trends of differential expression were retained in the Treatment vs. Case group. After obtaining the target miRNA, starBase was applied to predict the miRNA-lncRNA pairs, and the relationship pairs with opposite differential expression trends were retained as well. Thus, an mRNA-miRNA-lncRNA binding network was obtained for the Treatment vs. Case group.

Likewise, mirwalk was used to predict the target miRNAs of DE-ERSRG, and only miRNAs in the miRNAs-DE-ERSRG pairs with opposite trends of differential expressions were retained. After the miRNAs were obtained, the target circRNAs of the miRNAs were predicted using miRanda, and the pairs with the contrasting relationship of differential expression trends were selected. An mRNA-miRNA-circRNA network was established using the data.

### 2.8 Functional enrichment analyses of DE-ERSRGs in the ceRNA network

Gene Ontology (GO) and Kyoto Encyclopedia of Genes and Genomes (KEGG) enrichment analyses were performed with DE-mRNAs, mRNAs in the ceRNA and ceRNA (circ) networks as well as DE-ERSRGs from the three comparison groups individually using clusterProfiler (version4.0.2), with *p* < .05 as the selection standard.

### 2.9 Real-time qPCR

Eleven frozen samples were segregated into three groups, with three samples each in the control and case groups and five samples in the treatment group. Then, 50 mg of tissue was extracted from each sample and lysed using TRIzol Reagent (Life Technologies, CA, United States), and total RNA was isolated by following the manufacturer’s instructions. After the concentration and purity of RNA were determined, the RNA was reverse-transcribed to cDNA using the SureScript-First-strand-cDNA-synthesis-kit (Genecopoeia, Guangzhou, China) before qRT-PCR. The qRT-PCR mixture was composed of 3 µL of cDNA, 5 µL of 2x Universal Blue SYBR Green qPCR Master Mix (Servicebio, Wuhan, China), and 1 µL each of forward and reverse primers. PCR was performed in a BIO-RAD CFX96 Touch TM PCR detection system (Bio-Rad Laboratories, Inc., United States) under the following thermal cycling conditions: 40 cycles at 95°C for 60 s, 95°C for 20 s, 55°C for 20 s, and 72°C for 30 s. The 2^−△△Ct^ method was used to calculate gene expression levels, and GraphPad Prism 5 was applied to plot and calculate the statistical significance. The primer sequences used in this study are listed in Supplementary Table S3.

## 3 Results

### 3.1 Successful establishment of the chronic psychological stress model

Behavioral tests were performed 24 h after the end of stress induction in the three groups of mice. As shown in Supplementary Figure S2A, in the open-field experiment, mice from the case group spent significantly less time in the central zone than mice from the control and CB2R agonist treatment groups. Additionally, in the elevated plus maze test, the time of stay in the open arm in the case group was significantly lesser than that in the control group, but no difference was observed between the case and treatment groups (Supplementary Figure S2B). Furthermore, in the forced swimming test, the immobility time in the case group was significantly greater than that in the control and treatment groups (Supplementary Figure S2C). Finally, in the tail suspension test, the immobility time in the case group was significantly greater than that in the control group, but no statistically significant differences were observed compared with the treatment group (Supplementary Figure S2D). The above results indicated that the protocol could induce significant stress in the mice.

### 3.2 Identification of DE-mRNAs, DE-miRNAs, DE-lncRNAs, and DE-circRNAs

**FIGURE 1:**
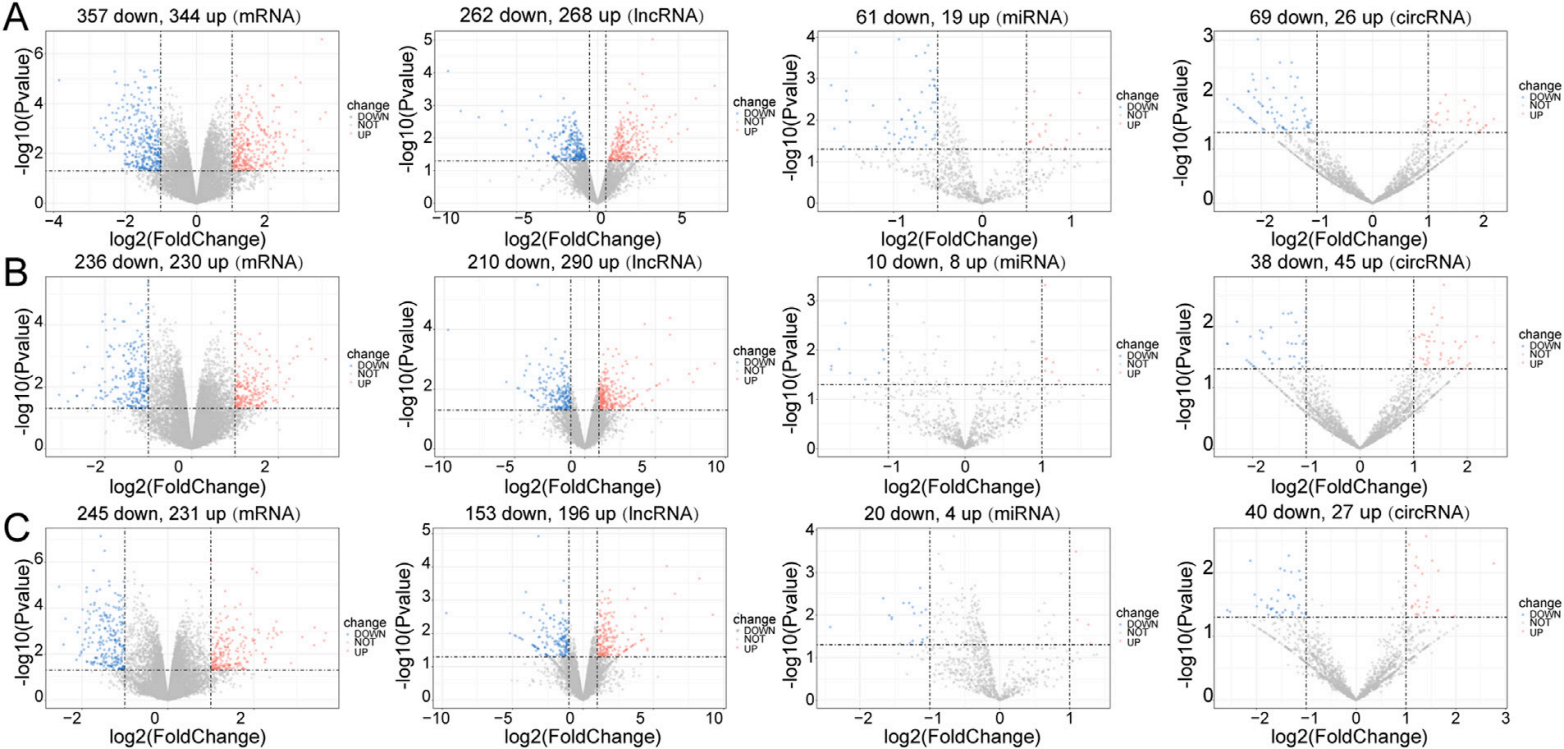
Differential expression analysis. Volcano plot for differentially expressed mRNAs (DE-mRNAs), lncRNAs (DE-lncRNAs), miRNAs (DE-miRNAs) and circRNAs (DE-circRNAs) in the three comparison groups. **(A)** Control vs. Case; **(B)** Case vs. Treatment; **(C)** Control vs. Treatment. Blue: downregulation, red: upregulation.

In the Control vs. Case group, 701 DE-mRNAs (357 upregulated, 344 downregulated), 80 DE-miRNAs (19 upregulated, 61 downregulated), 530 DE-lncRNAs (268 upregulated, 262 downregulated), and 95 DE-circRNAs (26 upregulated, 69 downregulated) were screened between four case mice and three normal case mice (Figure 1A). In the Treatment vs. Case group, 466 DE-mRNAs (230 upregulated, 236 downregulated), 18 DE-miRNAs (eight upregulated, ten downregulated), 500 DE-lncRNAs (290 upregulated, 210 downregulated), and 83 DE-circRNAs (45 upregulated, 38 downregulated) were obtained (Figure 1B). In the Control vs. Treatment group, 475 DE-mRNAs (231 upregulated, 245 downregulated), 24 DE-miRNAs (four upregulated, 20 downregulated), 349 DE-lncRNAs (196 upregulated, 153 downregulated), and 67 DE-circRNAs (27 upregulated, 40 downregulated) were eventually selected (Figure 1C).

In the Control vs. Case group, the majority of DE-mRNAs were located in the chromosomes, except the Y chromosome (only one downregulated DE-mRNA was located in Y chromosome). The DE-lncRNAs were almost evenly situated in each pair of chromosomes, and DE-miRNAs were situated in chromosomes 2, 3, 4, 7, 11, 12, 15, 17, 19, and X. DE-circRNAs were located in the euchromosomes and chromosome X (Supplementary Figure S3A).

In the Treatment vs. Case group, the DE-mRNAs, DE-lncRNAs, and DE-circRNAs were situated in all chromosomes except the Y chromosome. The DE-miRNAs were located in the 3, 5, 6, 7, 8, 11, 12, 13, 15, and X chromosomes (Supplementary Figure S3B).

In the Treatment vs. Control group, the locations of DE-mRNAs and DE-lncRNAs were similar to those in the Treatment vs. Case group, but the DE-circRNAs were located in the euchromosomes and Y chromosomes, and DE-miRNAs were located in the 1, 3, 5, 6, 7, 8, 10, 11, 12, 14, 15, 19, and X chromosomes (Supplementary Figure S3C).

### 3.3 Functional enrichment analyses of DE-mRNAs in the three groups

**FIGURE 2:**
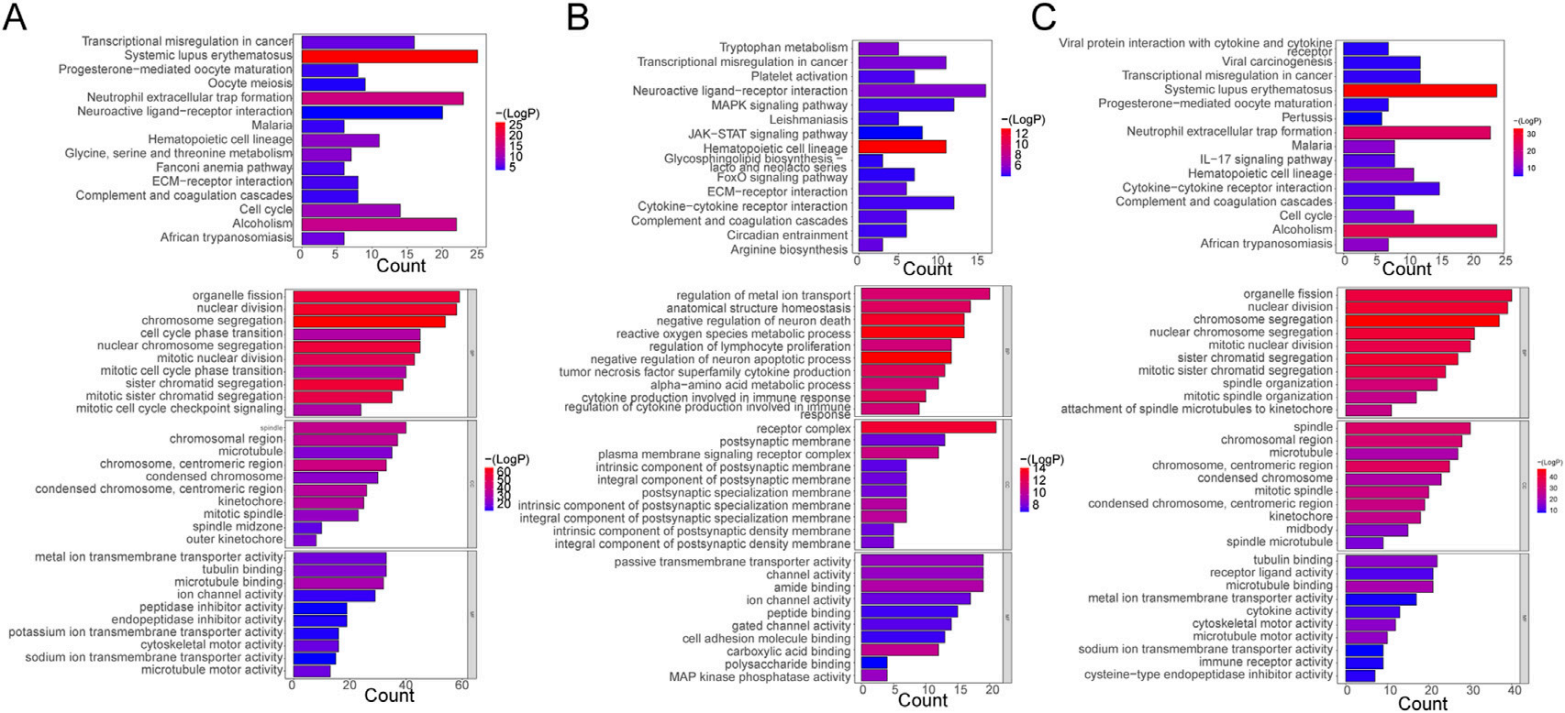
Functional Enrichment Analysis. **(A)** Bar plots of KEGG pathway and GO enrichment analyses in the control and case groups. **(B)** Bar plots of KEGG pathway and GO enrichment analyses in the case and treatment groups. **(C)** Bar plots of KEGG pathway and GO enrichment analyses in the control and case groups. Each GO map presents the top 10 enriched terms in BP, CC, and MF. Each KEGG map shows the top 15 enriched KEGG pathways.

The functional enrichment results of DE-mRNAs in the Control vs. Case group illustrated that 1261 GO terms and 98 KEGG pathways were enriched by 701 DE-mRNAs. For example, chromosome separation, nuclear division, nuclear chromosome segregation, organelle fission, and mitotic nuclear segregation were the major enriched GO BP terms. Chromosomes, centromeric region, spindle, and chromosomal region, among others, were the primarily enriched terms in CC. In MF, microtubule binding, tubulin binding, metal ion transmembrane transporter activity, microtubule motor activity, among others, were the major common terms. Furthermore, systemic lupus erythematosus, neutrophil extracellular trap formation, and alcoholism were the primarily enriched KEGG pathways (Figure 2A).

With respect to DE-mRNAs in the Treatment vs. Case group, 928 GO terms and 33 KEGG pathways were enriched by the 466 DE-mRNAs. The common enriched GO terms primarily included regulation of metal ion transport, anatomical structure homeostasis, negative regulation of neuron death, and others in BP; receptor complex, plasma membrane signaling receptor complex, integral component, and others in CC; and carboxylic acid binding, amide binding, kinase phosphatase activity, and others in MF. Additionally, hematopoietic cell lineage, neuroactive ligand-receptor interaction, transcriptional dysregulation in cancer, tryptophan metabolism, and others were the primarily enriched pathways (Figure 2B).

Furthermore, in the Treatment vs. Control group, the 476 DE-mRNAs enriched 952 GO terms. The major BP terms were chromosome segregation, nuclear division, sister chromatid segregation, nuclear chromosome segregation, and others. The major CC terms were chromosome, centromeric region, microtubule, chromosomal region, spindle, and others. The major MF terms were microtubule binding, receptor-ligand activity, tubulin binding, and others. Moreover, 31 KEGG pathways were enriched, and the top fifteen most significant pathways were shown in the chart, which primarily included alcoholism, neutrophil extracellular trap formation, and systemic lupus erythematosus, among others (Figure 2C).

### 3.4 ceRNA and ceRNA (circ) networks of the three groups

**FIGURE 3:**
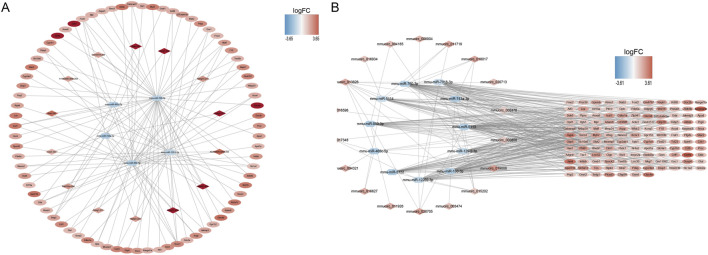
ceRNA network construction. **(A)** mRNA-miRNA-lncRNA network in control and case groups. **(B)** mRNA-miRNA-circRNA network in control and case groups. In the mRNA-miRNA-lncRNA (circRNA) network, the hexagon represents miRNA, the rhombus represents lncRNA (circRNA), the oval represents mRNA, and the color represents logFC value. The redder the color, the more upregulated the expression of the gene, and the bluer the color, the more downregulated the expression of the gene.

A ceRNA network containing 99 nodes and 127 edges and a ceRNA (circ) network containing 157 nodes and 354 edges were established using DE-mRNAs in the Case vs. Control group (Figure 3A, B). Based on the mRNAs in the Treatment vs. Case group, a ceRNA network with 104 nodes and 112 edges and a ceRNA (circ) network with 293 nodes and 492 edges were established (Supplementary Figures S4A, B). In the Treatment vs. Control group, a ceRNA network with 68 nodes and 66 edges and a ceRNA (circ) network with 226 nodes and 424 edges were established (Supplementary Figures S4C, D).

### 3.5 Functional enrichment analyses of mRNAs in the networks

**FIGURE 4:**
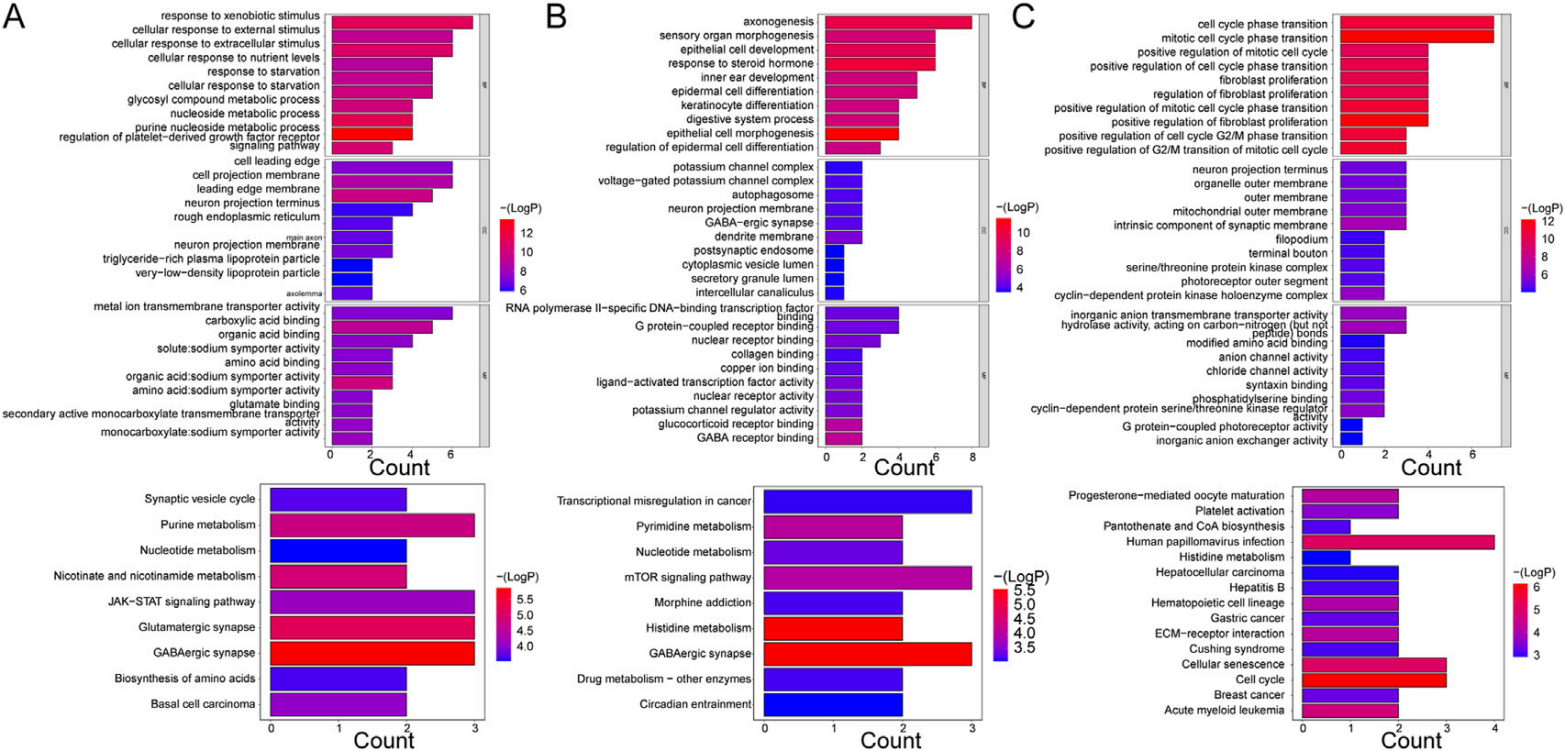
GO enrichment and KEGG pathway analyses of mRNAs enrolled in ceRNA networks. **(A)** KEGG pathway and GO enrichment analysis of mRNAs in the ceRNA (lncRNA) network in control and case groups. **(B)** KEGG pathway and GO enrichment analysis of mRNAs in the ceRNA (lncRNA) network in the case and treatment groups. **(C)** KEGG pathway and GO enrichment analysis of mRNAs in the ceRNA (lncRNA) network in the control and treatment groups. Each GO map shows the top 10 enriched phrases in BP, CC, and MF. On **(A, B)**, the top 9 enriched KEGG pathways are shown, while **(C)** shows the top 15 enriched KEGG pathways.

Five hundred and sixty-seven GO terms and nine KEGG pathways were enriched by the mRNAs in the ceRNA network of the Case vs. Control group. For instance, the purine nucleoside metabolic process, nucleoside metabolic process, response to xenobiotic stimulus, leading-edge membrane, cell projection membrane, cell leading edge, carboxylic acid binding, metal ion transmembrane transporter activity, and organic acid:sodium symporter activity were the primary enriched terms. The nine KEGG pathways were basal cell carcinoma, biosynthesis of amino acids, GABAergic synapse, glutamatergic synapse, JAK-STAT signaling pathway, nicotinate and nicotinamide metabolism, nucleotide metabolism, purine metabolism, and synaptic vesicle cycle (Figure 4A). Additionally, the mRNAs in the ceRNA (circ) network of the Case vs. Control group enriched 694 GO terms and 17 KEGG pathways, such as response to xenobiotic stimulus, small molecule catabolic process, cell projection membrane, and carboxylic acid binding in GO. Parathyroid hormone synthesis, secretion, and action, glyoxylate and dicarboxylate metabolism, breast cancer, and basal cell carcinoma were the major KEGG pathways (Supplementary Figure S5A).

With respect to the Case vs. Treatment group, the mRNAs in ceRNA network enriched axonogenesis, response to steroid hormone, epithelial cell development, dendrite membrane, G protein-coupled receptor binding, RNA polymerase II-specific DNA-binding transcription factor binding, and others among 426 GO terms. The nine enriched KEGG pathways primarily included GABAergic synapse, histidine metabolism, and mTOR signaling pathway (Figure 4B). In addition, the mRNAs in the ceRNA (circ) network commonly enriched 684 GO terms and 23 KEGG pathways, such as regulation of metal ion transport, glutamatergic synapse, carbohydrate binding, transcriptional dysregulation in cancer, and FoxO signaling pathway (Supplementary Figure S5B).

Finally, in the Treatment vs. Control group, the mRNAs from the ceRNA network enriched 314 GO terms and 14 KEGG pathways. The enriched BP terms were more significant than the CC and MF terms, such as mitotic cell cycle phase transition, cell cycle phase transition, and positive regulation of fibroblast proliferation. The most significantly enriched pathways included cell cycle, cellular senescence, and human papillomavirus infection, among others (Figure 4C). Besides, the enrichment results of mRNAs in the ceRNA (circ) network were similar in that the enriched BP terms were more significant than those in CC and MF as well, such as regulation of body fluid levels, G2/M transition of mitotic cell cycle, and cell cycle G2/M phase transition. Hematopoietic cell lineage, cytokine-cytokine receptor interaction, *Staphylococcus aureus* infection, and others were some of the primarily enriched KEGG pathways (Supplementary Figure S5C).

### 3.6 Identification and functional annotation of DE-ERSRGs

**FIGURE 5:**
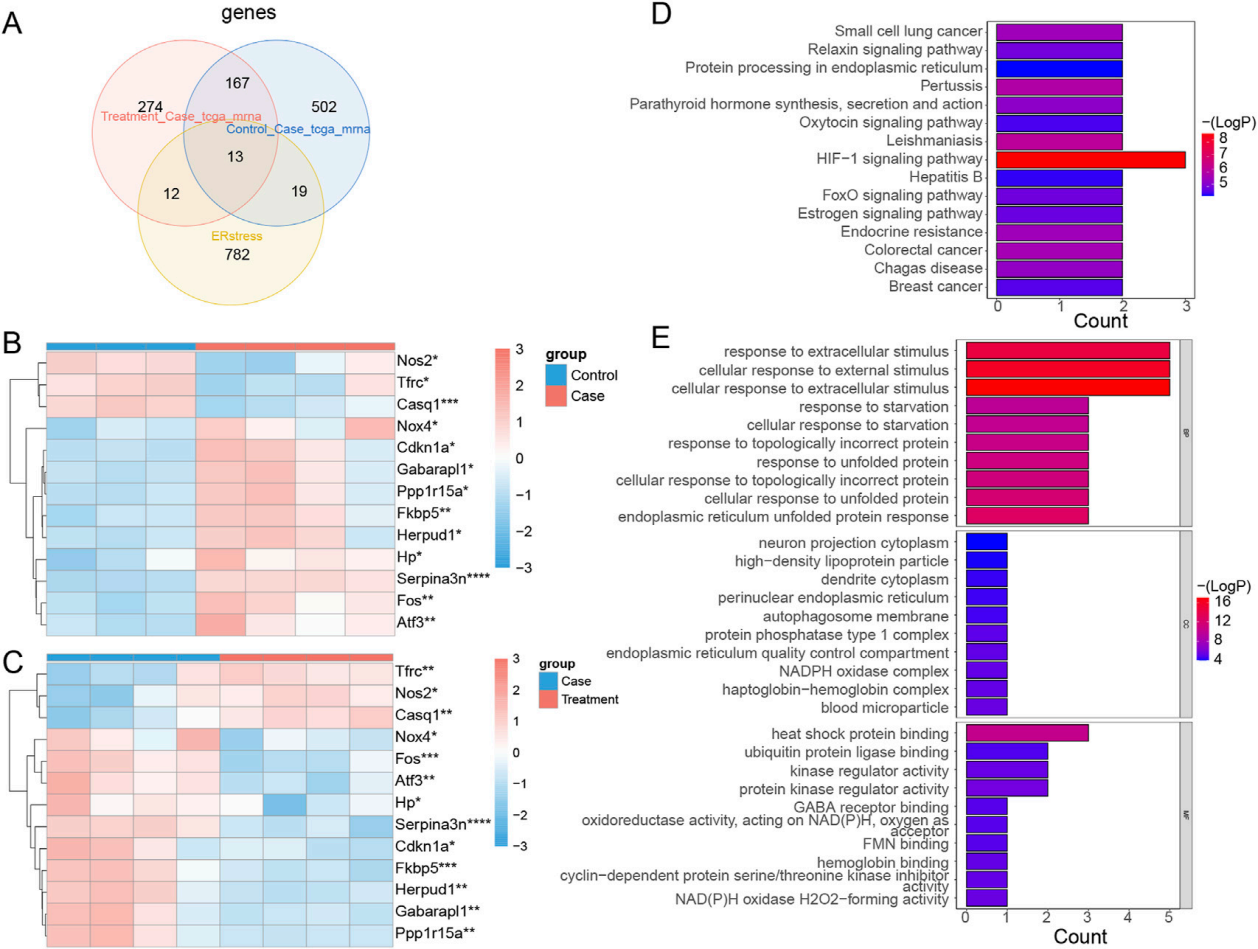
Identification of DE-ERSRGs and functional enrichement analysis. **(A)** Venn diagram of 13 DE-ERSRGs by overlapping DEGs in control vs. case group, DEGs in case group vs. treatment, with 826 ER stress-related genes. **(B)** Heat map of expression of 13 DE-ERSRGs in control and case groups. **(C)** Heat map of expression of 13 DE-ERSRGs in the case and treatment groups. **(D)** KEGG pathway analysis of 13 DE-ERSRGs. **(E)** GO enrichment analysis of 13 DE-ERSRGs.

Thirteen intersected mRNAs (Fos, Hp, Serpina3n, Cdkn1a, Atf3, Nos2, Fkbp5, Tfrc, Nox4, Gabarapl1, Herpud1, Casq1, and Ppp1r15a) were identified from the overlap analysis; these were considered DE-ERSRGs (Supplementary Table S4; Figure 5A). The expression levels of DE-ERSRGs in the Case group were significantly different from those in the Control groups (Supplementary Figure S6A). Of note, the expression of Nos2, Tfrc, and Casq1 was higher in the control group (Figure 5B). Furthermore, these DE-ERSRGs were expressed differently between the Treatment and Case groups as well (Supplementary Figure S6B), and the expression levels of Tfrc, Nos2, and Casq1 were significantly higher in the treatment group (Figure 5C).

Moreover, the enrichment results indicated that 24 KEGG and 429 GO term pathways were enriched by the 13 DE-ERSRGs. HIF-1 signaling was the most significantly enriched KEGG pathway, followed by Leishmaniasis, Pertussis, and others (Figure 5D). Furthermore, cellular response to extracellular stimulus, cellular response to external stimulus, response to extracellular stimulus, and other terms, were the primary BP terms. Meanwhile, the CC terms included dendrite cytoplasm, high-density lipoprotein particle, neuron projection cytoplasm, and others. Heat shock protein binding, ubiquitin protein ligase binding, kinase regulator activity, and others were the primary terms in MF (Figure 5E).

### 3.7 Establishment of DE-ERSRG-based ceRNA and ceRNA (circ) networks

**FIGURE 6:**
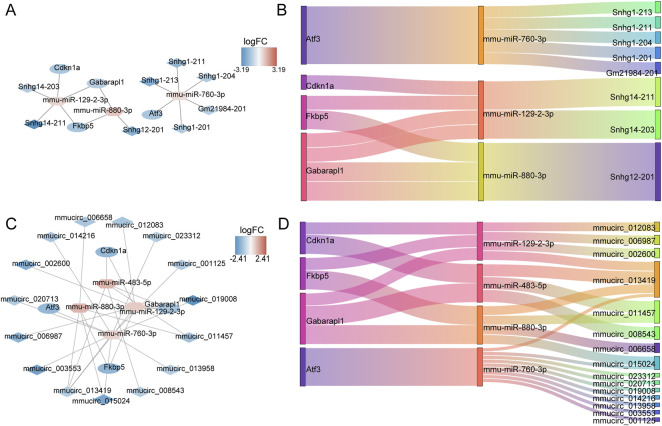
Construction of ceRNA networks for 13 DE-ERSRGs. **(A)** mRNA-miRNA-lncRNA regulatory network. **(B)** Sankey diagram of the mRNA-miRNA-lncRNA regulatory. **(C)** mRNA-miRNA-circRNA regulatory network. **(D)** Sankey diagram of mRNA-miRNA-circRNA regulatory. In the mRNA-miRNA-lncRNA (circRNA) network, the hexagon represents miRNA, the rhombus represents lncRNA (circRNA), the oval represents mRNA, and the color represents logFC value. The closer the color is to red, the more upregulated the expression is, and the closer the color is to blue, the downregulated the expression is.

The ceRNA network of 13 DE-ERSRGs illustrated that 15 nodes and 14 edges were present in the network, which only contained four of the 13 DE-ERSRGs (Cdkn1a, Atf3, Fkbp5, and Gabarapl1) (Figures 6A, B). In addition, a ceRNA (circ) network with 23 nodes and 28 edges was established based on the 13 DE-ERSRGs, which only contained the same four DE-ERSRGs (Figures 6C, D). Therefore, these four genes were regarded as key DE-ERSRGs. Similarly, according to the online dataset from GEO database (GSE210252), the expression patterns of four key DE-ERSRGs between mice received restraint stress or no stress were further explored and exhibited in Supplementary Figure S7, supporting that Cdkn1a, Atf3, and Gabarapl1 expressed higher in case group than that in control group, which were consistent to the results of our study above that shown in Supplementary Figure S6A.

### 3.8 RT-qPCR validation of 13 DE-ERSRGs

**FIGURE 7:**
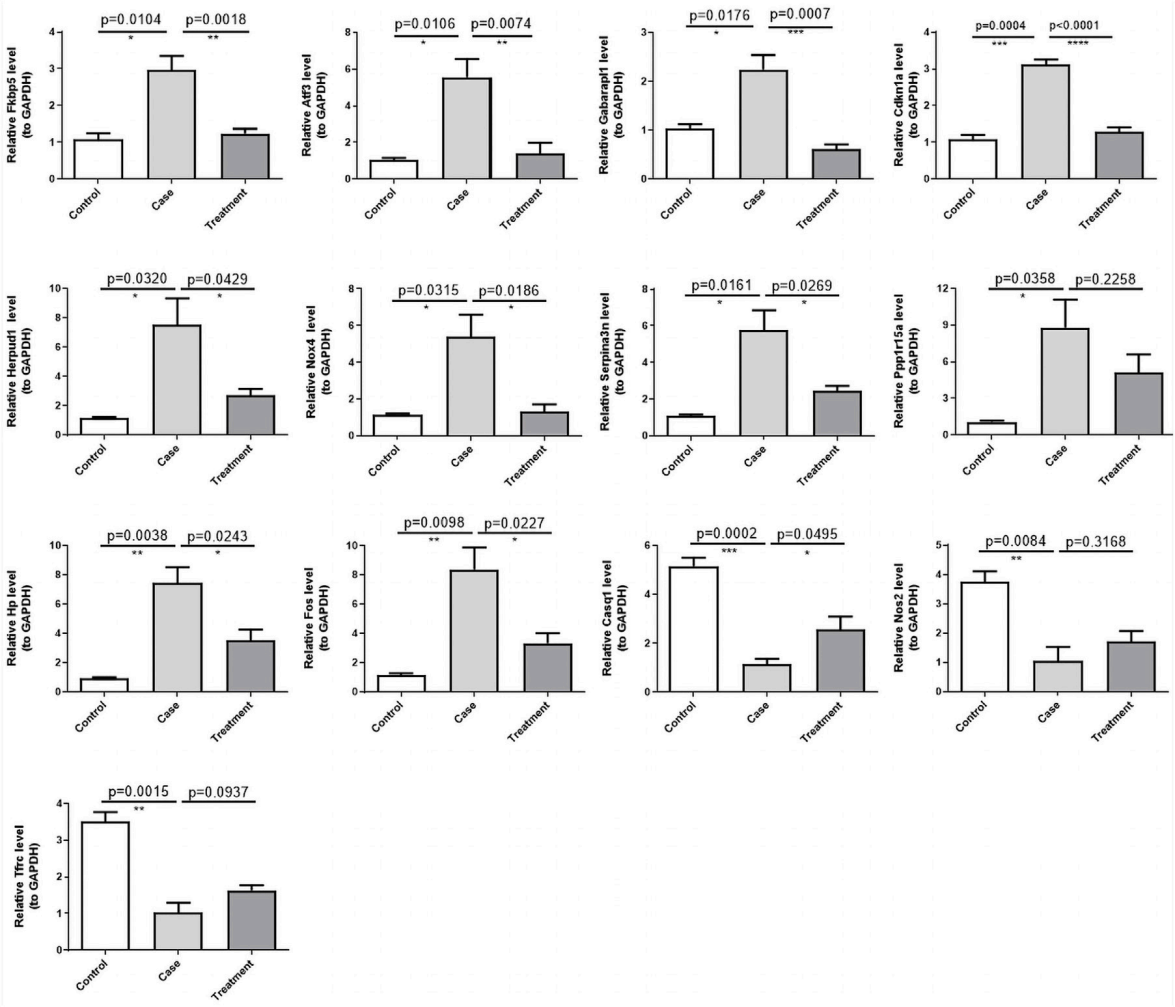
Validation of the expression of 13 DE-ERSRGs by RT-qPCR. Validation of the expression levels of 13 DE-ERSRGs in mice tissue samples by quantitative RT-PCR. Error bars show mean ± SD. Data are representative of three independent experiments. *p < .05, **p < .01, ***p < .001.

To gain deeper insights on the expression levels of the 13 DE-ERSRGs (Fos, Hp, Serpina3n, Cdkn1a, Atf3, Nos2, Fkbp5, Tfrc, Nox4, Gabarapl1, Herpud1, Casq1, and Ppp1r15a), RT-qPCR was performed using eleven tissue samples. The expression levels of the DE-ERSRGs were distinctly different between the control and case samples (all *p* < .05), which was consistent with the results of previous bioinformatics analysis. Conversely, the expression levels in the case and treatment groups were significantly different, except for the levels of Ppp1r15a and Nos2. Although the expression levels of these two genes were not statistically significant, they were altered, as confirmed by the sequencing results (Figure 7).

## 4 Discussion

Numerous studies, including epidemiological and experimental studies in humans and animals, have yielded compelling evidence favoring the strong association between psychosocial factors and cardiovascular morbidity. In current European guidelines for the prevention of CVDs, psychological stress is recognized as a potential contributing factor to both CVD development and progression (Piepoli et al., 2016). Yet, the limited understanding of the molecular mechanisms underpinning chronic stress-induced cardiac injury has led to the lack of effective treatment options. Several studies have shown that psychological stress is a determinant of the prognosis and outcome of cardiovascular events, rather than a cause of CVD (Sara et al., 2021). On one hand, psychological stress can contribute to some adverse lifestyle patterns in humans, such as the abuse of tobacco and alcohol, poor eating habits, or sleep disorders. These confounding factors complicate the assessment of the effects of mental factors on the cardiovascular system in a given context. On the other hand, in animal experiments, subjects suffering from CVDs were simultaneously exposed to psychological stress to investigate the effect of stress on the progression of pre-existing CVDs, but limited attention was paid to the potential molecular processes induced by chronic psychological stress that induce cardiac tissue damage in healthy individuals. Cho et al. evaluated the molecular basis of cardiac damage in healthy persons exposed to psychological stress using a transcriptome sequencing approach, but their results failed to adequately account for the irreversible damage caused to the heart by chronic stress in American soldiers (Cho et al., 2014). This might be related to the methods (social defeat) they used, because the recurrence of the same stressor usually leads to adaptation and failure to reflect the unpredictable, uncontrollable, complex, and variable properties of naturally occurring stressors. In our study, we addressed and resolved some of the limitations of their study. The chronic psychological stress model was slightly modified from the chronic unpredictability stress model by increasing the stimulus intensity to reduce adaptation and better simulate chronic stress. The results suggested that mice that were subjected to the stress stimuli exhibited significant differences in all four behavioral experiments compared to control mice. In this modified model, we observed the abnormal expression of some ERSRGs, and the occurrence of ER stress may be a cause of permanent cardiac damage in healthy individuals suffering from chronic psychological stress.

In recent years, a large body of evidence from research has shown that ER stress is linked to the pathogenesis and progression of multiple CVDs and is an important target for the treatment of diverse CVDs. In this study, we identified 13 DE-ERSRGs. Among them, Fos was previously reported to be a downstream signaling molecule of ER stress in obesity models (Levi et al., 2018), and GRP78 was shown to interact with the transcription factor Fos to promote ER stress (Gopal and Pizzo, 2021). Fu et al. found that the overexpression of sarcoplasmic ER calcium ATPase (SERCA2a) reduced Hp expression while alleviating ER stress, thereby improving cardiac function in animals with heart failure. This suggests that Hp expression may be positively correlated with the ER stress levels in cardiac tissues (Fu et al., 2012). Serpina3 has been detected as a marker of ER stress in astrocytes in immune system diseases (Masvekar et al., 2022), and the role played by its Serpina3n (a protein from the same family) in ER stress has never been described. The role of NOX4, a member of the NADPH oxidase family, in ER stress in CVDs has garnered greater attention, as NOX4 participates in two UPR signaling pathways (Wu et al., 2010). In vascular smooth muscle cells and cardiomyocytes ontogenically related to VSMCs, high NOX4 expression induced by ER stress reduced cell death but promoted cell proliferation, leading to arterial remodeling and myocardial hypertrophy, and exacerbated the deterioration of cardiac function (Mittal et al., 2007; Kuroda et al., 2010). The PP1-Ppp1r15a phosphatase complex dephosphorylates eIF2α, and the anti-hypertensive compound guanabenz disrupts this complex and thereby attenuates ER stress in cardiomyocytes (Neuber et al., 2014). We also detected the upregulation of this complex in cardiac tissues post stress, which was reduced upon treatment with CB2R agonists. Herpud1 is an ER-resident membrane protein that plays a part in ER degradation, and its upregulation can exert a cytoprotective role, which is why we assumed that the upregulation of Herpud1 in this experiment is a compensatory protective mechanism (Navarro-Marquez et al., 2018). Furthermore, three DE-ERSRGs in our results showed a trend of downregulation in response to psychological stress and upregulation after treatment. Among them, Tfrc and Casq1 usually play a protective role and lead to the generation of ER stress when external factors cause their lowering (Jung et al., 2015; Hanna et al., 2021), which is also consistent with our results. Payne et al. suggested that Nos2 could protect against the development of ER stress (Payne et al., 2001), whereas in other studies, increased Nos2 expression has been suggested to exacerbate ER stress (Deslauriers et al., 2011; Zanotto et al., 2017). Whether the tendency of Nos2 downregulation in response to psychological stress results from a compensatory mechanism or some other mechanism remains elusive. Cdkn1a has been reported in ER stress (Lopez et al., 2015; Inoue et al., 2017). In this model, Cdkn1a probably acted as a negative regulator of the cell cycle, allowing cells sufficient time for repair of damaged DNA after external stimuli and various metabolic abnormalities. Moreover, Gabapal1 is involved in the formation of autophagosomal vacuoles (Chakrama et al., 2010). We hypothesized that it may participate in ER stress-induced autophagy. Atf3 is involved in ER stress through multiple pathways. In the most common pathway, Atf3 acts as an Atf4 downstream molecule in PERK-ATF4-ATF3-CHOP-induced apoptosis (Tang et al., 2020). In addition, Atf3 can also act as a transcription factor for NOX4 and upregulate NOX4-induced ROS production (Liu Z. et al., 2020). This connection can also explain the increased Nox4 expression observed in our results. Additionally, Fkbp5 is a well-known stress response gene that plays a central role in psychological stress-induced diseases (Le-Niculescu et al., 2020). The increase in Fkbp5 expression also corroborates the validity of our established model of chronic psychological stress. Targeted drugs of FKBP5 are being developed actively, and CB2R agonists may be one of the promising candidates.

Among the 13 DE-ERSRGs, four key gene could constitute the ceRNA regulative network. They have been reported to be strongly associated with the onset and progression of CVDs. The protein p21 encoded by cdkn1a is considered to be a marker of cardiomyocyte senescence in addition to inhibiting the cell cycle (Bernard et al., 2020). By increasing the levels of p21 intracellularly in cardiac myocytes, phenylalanine causes premature cardiomyocyte senescence (Czibik et al., 2021). Additionally, p21 overexpression in cardiac fibroblasts induces differentiation of primary cardiac fibroblasts into myofibroblasts and promotes cardiac remodeling (Roy et al., 2007). In a pressure overload-induced cardiac remodeling paradigm, cdkn1a knockdown mice showed reduced myocardial hypertrophy and fibrosis and improved cardiac function when compared to wild type mice (Xu and Tang, 2016). It is evident that cdkn1a is crucial in cardiac injury. Acute myocardial infarction (AMI) can induce the release of gabarapl1 from circulating endothelial cells, which can stimulate an increase in monocytes and neutrophils by recruiting NLRP3 inflammasomes, finally causing vascular inflammation during AMI (Zhang et al., 2022). ATF3, which functions as a “hub” for numerous adaptive cellular responses, is important for the progression and development of CVDs. ATF3 overexpression in cardiac tissues can promote myocardial hypertrophy and fibrosis (Okamoto et al., 2001). Elevated ATF3 expression due to different stimuli can damage endothelial cells to promote atherosclerosis (Cai et al., 2000). ATF3 was increased in carotid artery of hypertensive rats, and downregulation of ATF3 was associated with the protective effect of enalapril against hypertension (Liu et al., 2015). However, ATF3 plays a paradoxical role in the development of heart failure (Lin et al., 2014; Brooks et al., 2015). Thus, the role of ATF3 in CVDs is complicated. FKBP5 is a key molecule in the triggering of CVDs by psychological stress. FKBP5 can increase plaque destabilization by promoting local inflammation *via* the NF-κB pathway, according to previous research (Zannas et al., 2019). High levels of FKBP5 expression were also detected in platelets after AMI (Eicher et al., 2016). FKBP5 single nucleotide polymorphisms (SNPs) are closely related with depression and coronary heart disease co-morbidity (Wang et al., 2020).

Cho et al. assessed the dynamic course of gene expression in the mouse heart in the transcriptional level analysis after 1–10 days of exposure to social defeat (Cho et al., 2014). They identified extracellular matrix remodeling, immune responses (e.g., complement activation), and cell proliferation as the primary molecular-level alterations in cardiac tissues after psychological stress. Despite the differences in the models (social defeat vs. modified CMS), sequencing methods (microarray vs. whole transcriptome sequencing), and durations of stress (10 days vs. 3 weeks), we observed similar changes in gene expression patterns by analyzing the KEGG and GO enrichment results, suggesting that the same pathophysiological changes mentioned above were the basic reactions to psychological stress affecting the heart. In addition, we observed some notable changes, such as metabolic alterations in tissues (including glycine, serine, and threonine metabolism, purine metabolism, nucleotide metabolism, and nicotinate and nicotinamide metabolism.) and ER stress, which was the focus of this study.

Notably, the use of CB2R agonists diminishes the severity of stress in mice, as seen in the behavioral results, although there was no statistically significant difference between the results of the elevated plus maze and tail suspension tests, and only a trend toward improvement was observed. Possibly, ER stress occurs in neurons in regions such as the hippocampus, amygdala, and striatum, which are engaged in the development of psychological stress (Pavlovsky et al., 2013; Jangra et al., 2016; Jangra et al., 2017; Liu et al., 2019). CB2R may attenuate the symptoms of psychological stress by reducing the expression of ERSRGs and other pathways. CB2R agonists have been reported to exert neuroprotective effects to counteract the symptoms of psychological stress through anti-inflammatory functions (Zoppi et al., 2014).

However, there remain some limitations in our study. First, the sample size was small, which could have led to the low concordance between the qRT-PCR and sequencing results. Second, the experimental subjects used were male mice, which might have introduced gender bias. Third, the sequencing of whole heart tissues may have masked specific pathological changes in a particular region. We intend to follow up with experimental validation of the regulatory network of the four key genes identified and the role of CB2R agonists. We will also focus on the impact of metabolite changes in the results. In summary, our findings provide a comprehensive overview of the molecular changes in post-transcriptional regulation underlying chronic psychological stress-induced cardiac injury, with ER stress as one of the important mechanisms. CB2R agonists can alleviate ER stress through a competitive endogenous RNA mechanism and thus can be used as a therapeutic target in chronic psychological stress-induced cardiac injury.

## 5 Conclusion

Our findings revealed, for the first time, the expression profiles of circRNA, lncRNA, miRNA, and mRNA in cardiac tissues exposed to chronic psychological stress. We identified four key DE-ERSRGs-related genes and established relevant ceRNA networks. Our findings may provide novel insights on the molecular mechanisms underlying the effects of CB2R agonists in the treatment of chronic psychological stress-induced cardiac diseases.

## Data Availability

The datasets presented in this study can be found in online repositories. The names of the repository/repositories and accession number(s) can be found below: https://www.ncbi.nlm.nih.gov/geo/, GSE210820; https://www.ncbi.nlm.nih.gov/geo/, GSE210252.
