# Supplementary material for: Transcriptome-wide analysis reveals the molecular mechanisms of cannabinoid type II receptor agonists in cardiac injury induced by chronic psychological stress
Source: Front Genet. 2023 Jan 10;13:1095428. doi: 10.3389/fgene.2022.1095428 (PMC9871316; doi:10.3389/fgene.2022.1095428)
Supplement: Supplementary file 1 [file DataSheet1.zip › Supplementary Material_Revised_Manuscript_1095428.docx]

Supplementary Material

**
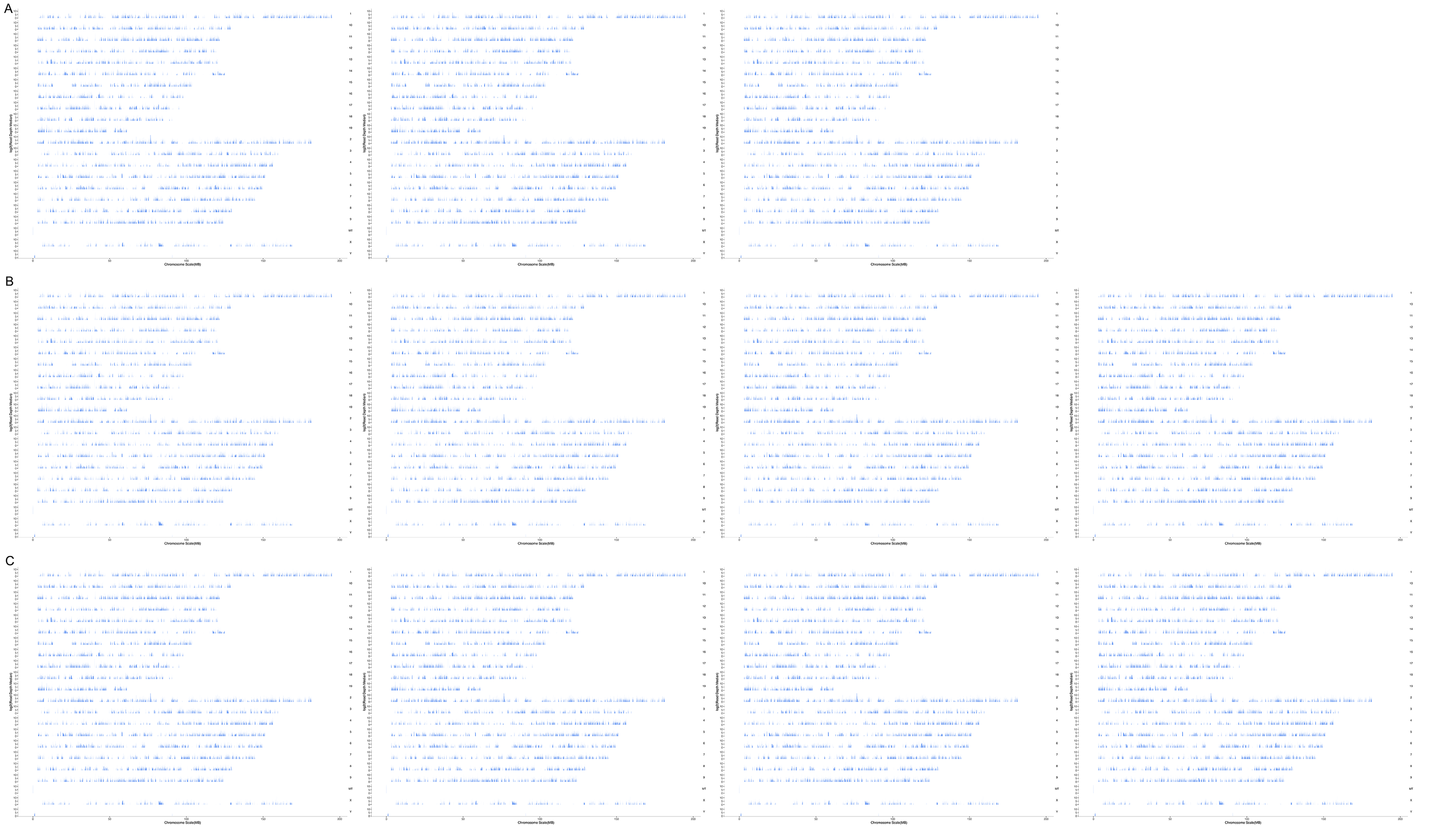
Supplementary Figure 1.** The sequencing depth in the ranscriptome-wide analysis for each sample from the three comparison groups. (A) The sequencing depth information of three normal samples. (B) The sequencing depth information of four case samples with psychological stress-induced heart disease. (C) The sequencing depth information of four treatment samples with psychological stress-induced heart disease treated with the CB2R agonist JWH133.


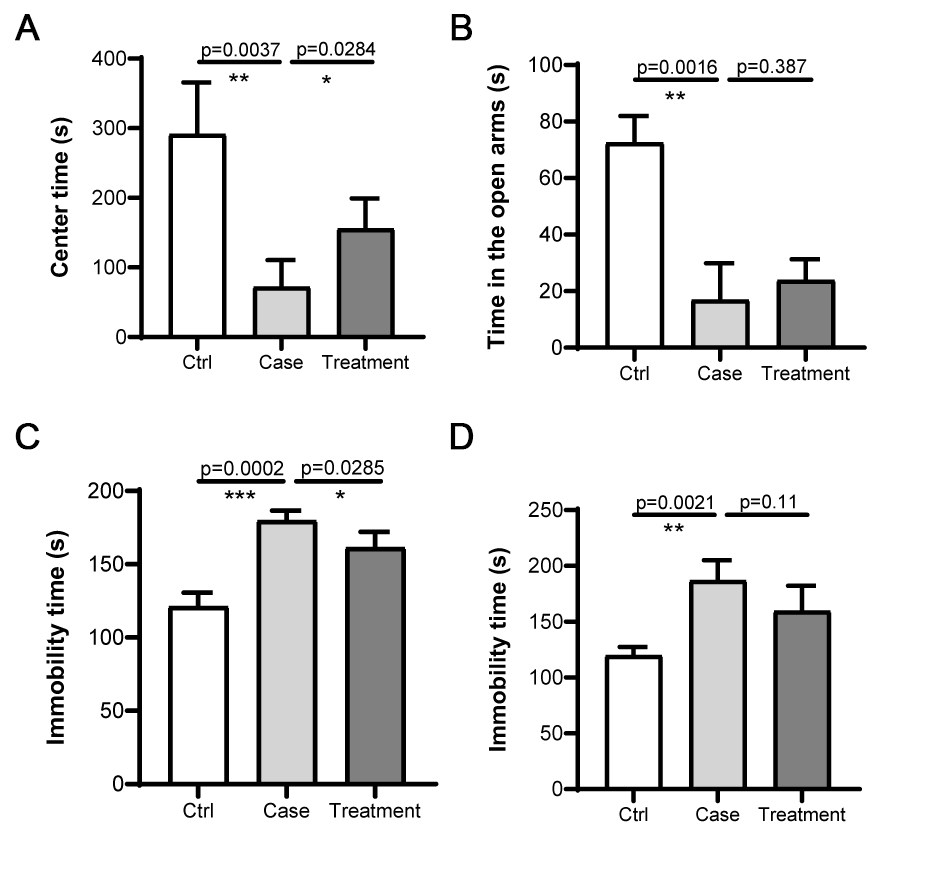


**Supplementary Figure 2.** Chronic psychological stress model was successfully established. Behavioral analysis of control, case and treatment group using open field test(A), elevated plus maze (B), forced swimming test(C)and tail suspension test(D). Number of mice used in behavior tests: Control group, Ctrl, n = 3 mice; Case group, Case, n = 4 mice; Treatment group, Treatment, n = 4 mice; Data represent mean ± SD; *p < 0.05, **p < 0.01, ***p < 0.001.





**Supplementary Figure 3.** Distribution of differentially expressed (DE) genes on chromosomes. (A) Gene circle plots of DE-mRMAs, DE-lncRNAs, DE-miRNAs, and DE-circRNAs in the control and case groups. (B) Gene circle plots of DE-mRMAs, DE-lncRNAs, DE-miRNAs, and DE-circRNAs in the case and treatment groups. (C) Gene circle plots of DE-mRMAs, DE-lncRNAs, DE-miRNAs, and DE-circRNAs in the control and treatment groups.


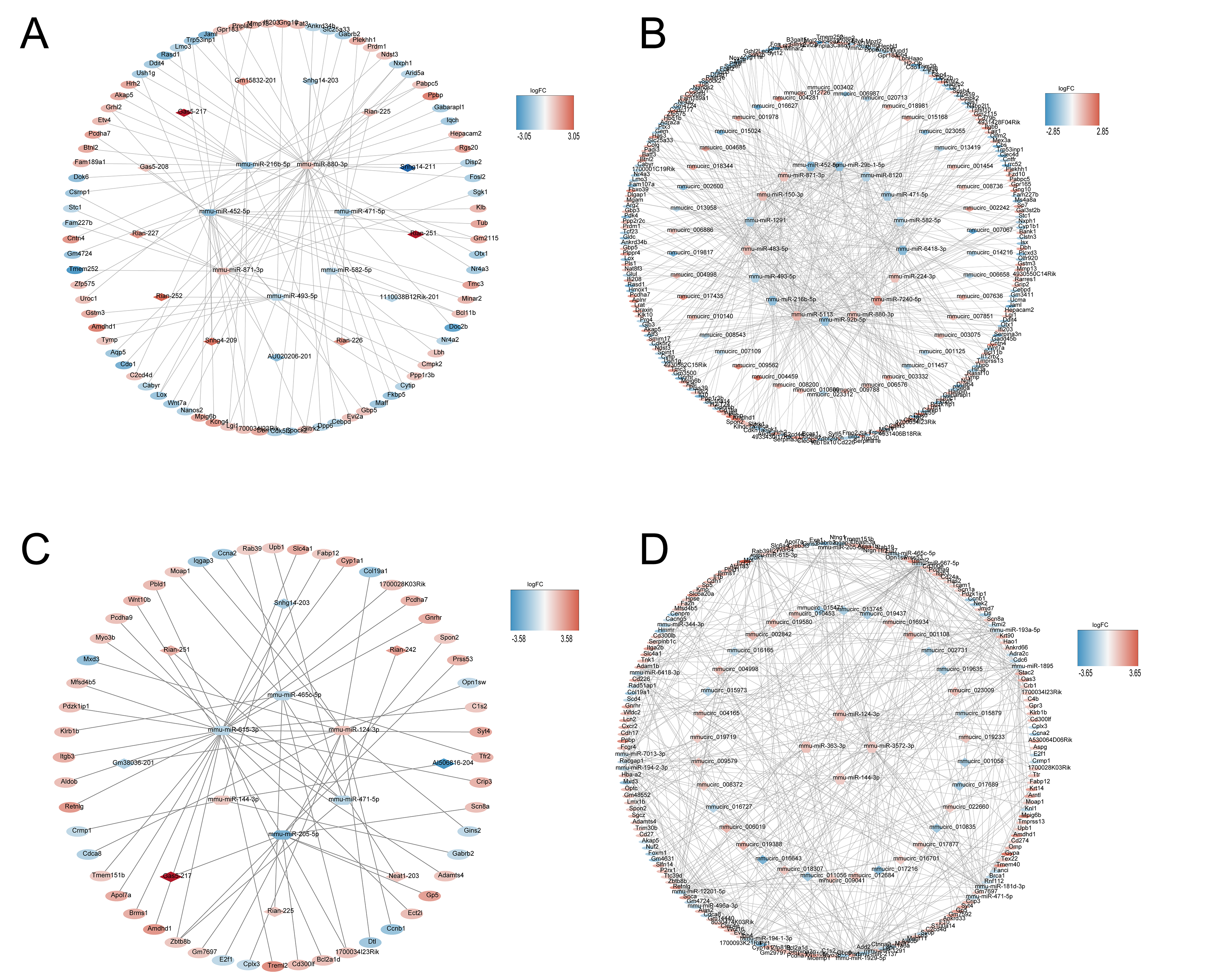


**Supplementary Figure 4.** ceRNA network construction. (A) The mRNA-miRNA-lncRNA network in the case and treatment groups. (B) The mRNA-miRNA-circRNA network in the case and treatment groups. (C)mRNA-miRNA-lncRNA network in the control and treatment groups. (D)The mRNA-miRNA-circRNA network in the control and treatment groups. In the mRNA-miRNA-lncRNA(circRNA) network, the hexagon represents miRNA, the rhombus represents lncRNA(circRNA), the oval represents mRNA, and the color represents logFC value. The closer the color is to red, the more up-regulated the expression is, and the closer the color is to blue, the down-regulated the expression is.


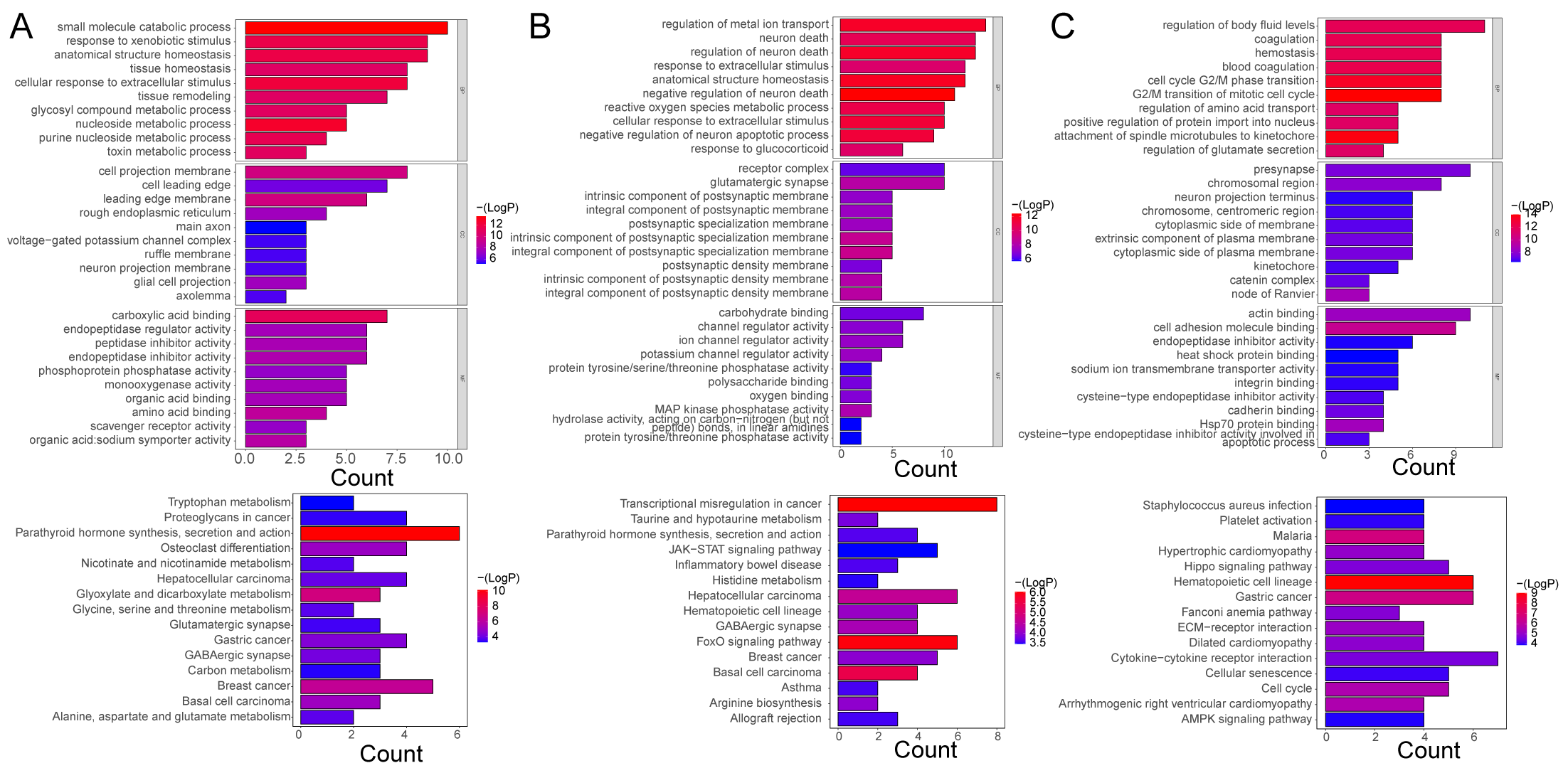


**Supplementary Figure 5.** Analysis of mRNA enrichment in ceRNA networks**.** (A) Top 150 KEGG pathways and top 30 GO terms of mRNAs in the ceRNA (circRNA) network in control and case groups. (B) Top 15 KEGG pathways and top 30 GO terms of mRNAs in the ceRNA (circRNA) network in the case and treatment groups. (C)Top 15 KEGG pathways and top 30 GO terms of mRNAs in the ceRNA (circRNA) network in control and treatment groups.


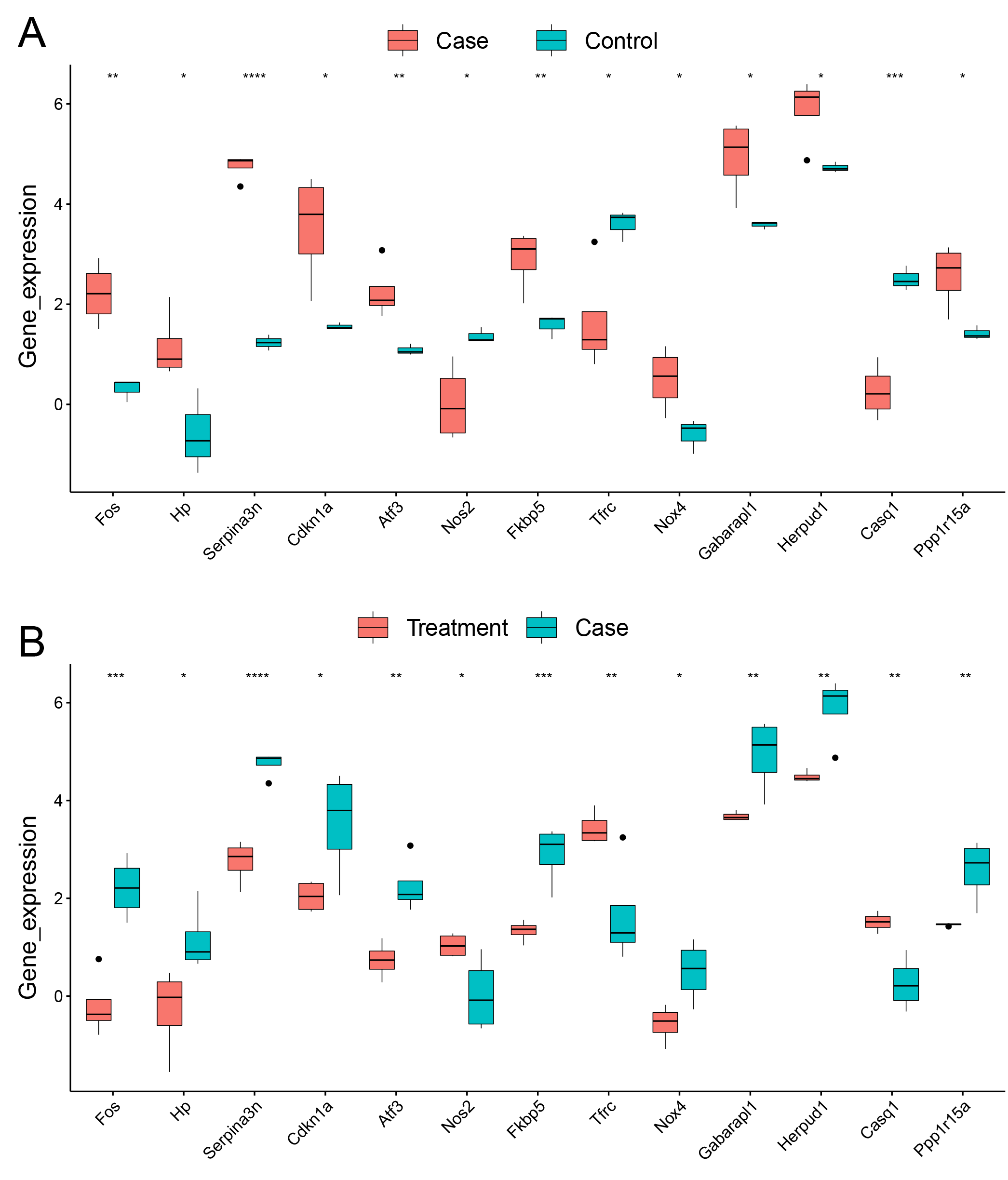


**Supplementary Figure 6.** The expression levels of the 13 DE-ERSRGs. (A) Box plot of the expression difference of DE-ERSRGs between control and case groups. (B) Box plot of the expression difference of DE-ERSRGs between the case and treatment groups. * represents P<0.05, **, represents P<0.01, *** represents P<0.001, and **** represents P<0.0001.


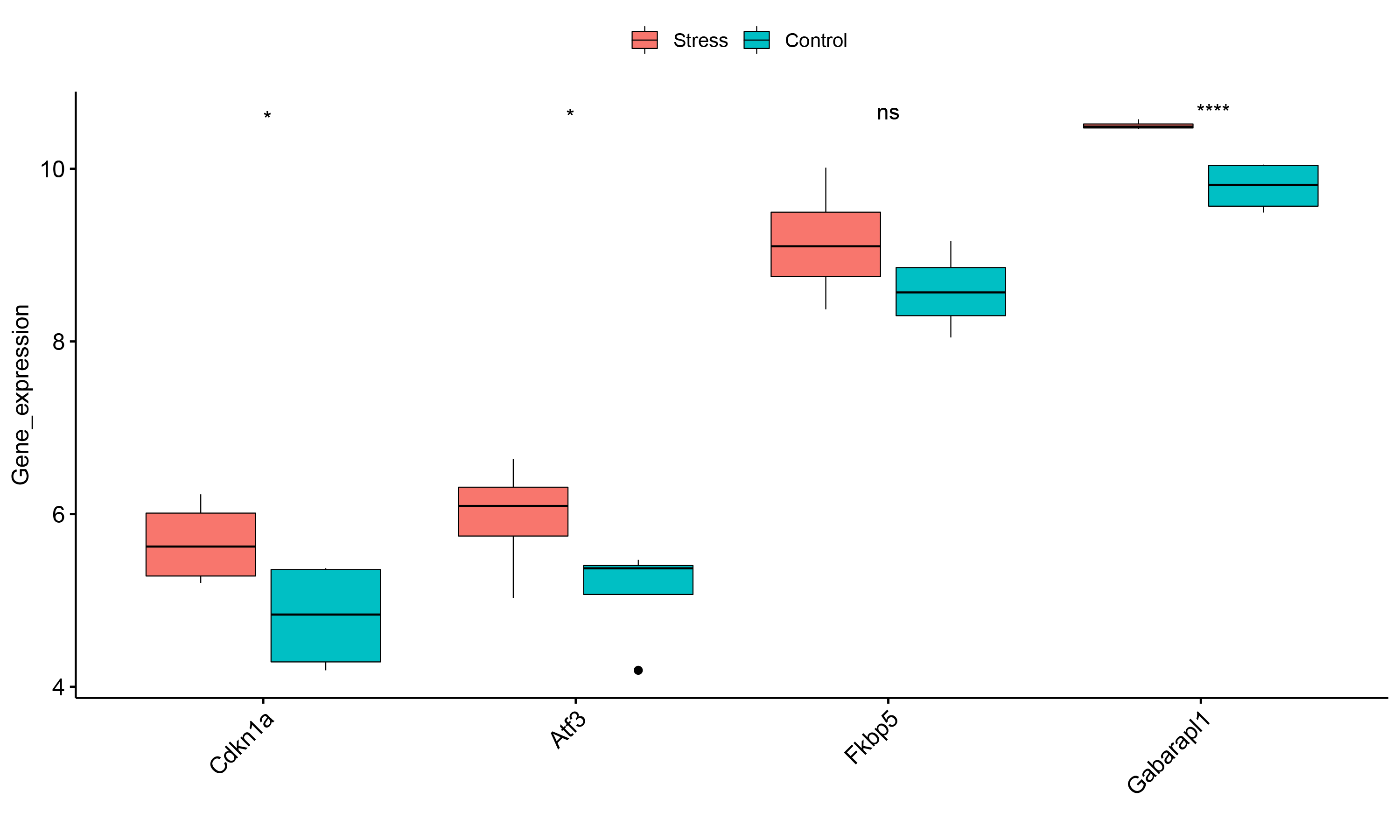


**Supplementary Figure 7.** The expression levels of four key DE-ERSRGs in GSE210252 dataset.

**Supplementary Table 1.** Chronic psychological stress procedure

**Supplementary Table 2.** 828 ERS-related genes in mice.

|  | HGNC.symbol | MGI.symbol |  | HGNC.symbol | MGI.symbol |  | HGNC.symbol | MGI.symbol |
| --- | --- | --- | --- | --- | --- | --- | --- | --- |
| 1 | PRKAA1 | Prkaa1 | 277 | IREB2 | Ireb2 | 553 | CYBB | Cybb |
| 2 | CDH2 | Cdh2 | 278 | ERLIN1 | Erlin1 | 554 | ERLIN2 | Erlin2 |
| 3 | TTR | Ttr | 279 | PARP1 | Parp1 | 555 | G6PD | G6pd2 |
| 4 | GSK3B | Gsk3b | 280 | TXNDC12 | Txndc12 | 556 | P4HB | P4hb |
| 5 | HSP90AA1 | Hsp90aa1 | 281 | CDKAL1 | Cdkal1 | 557 | PLA2G6 | Pla2g6 |
| 6 | SREBF2 | Srebf2 | 282 | CLGN | Clgn | 558 | CDC42 | Cdc42 |
| 7 | DSC2 | Dsc2 | 283 | FBXO6 | Fbxo6 | 559 | ATP2C1 | Atp2c1 |
| 8 | LPIN1 | Lpin1 | 284 | CRYAB | Cryab | 560 | APOE | Apoe |
| 9 | DERL1 | Derl1 | 285 | UBE4B | Ube4b | 561 | FPR2 | Fpr2 |
| 10 | SOD1 | Sod1 | 286 | EMD | Emd | 562 | FPR2 | Fpr3 |
| 11 | DSG2 | Dsg2 | 287 | MAPK10 | Mapk10 | 563 | JUP | Jup |
| 12 | DSG3 | Dsg3 | 288 | TRAM1 | Tram1 | 564 | COL9A2 | Col9a2 |
| 13 | RELA | Rela | 289 | TLR9 | Tlr9 | 565 | CES1 | Ces1b |
| 14 | RAB1B | Rab1b | 290 | MAN2A1 | Man2a1 | 566 | CES1 | Ces1c |
| 15 | PKP2 | Pkp2 | 291 | ADIPOQ | Adipoq | 567 | CES1 | Ces1d |
| 16 | MAPK1 | Mapk1 | 292 | DMD | Dmd | 568 | CES1 | Ces1g |
| 17 | SIL1 | Sil1 | 293 | AKAP9 | Akap9 | 569 | CES1 | Ces1h |
| 18 | SSR4 | Ssr4 | 294 | RTN4 | Rtn4 | 570 | RNF186 | Rnf186 |
| 19 | ADRB2 | Adrb2 | 295 | SURF4 | Surf4 | 571 | CR2 | Cr2 |
| 20 | SERPINI1 | Serpini1 | 296 | SERP2 | Serp2 | 572 | SEC62 | Sec62 |
| 21 | CASP7 | Casp7 | 297 | CYB5A | Cyb5a | 573 | POMC | Pomc |
| 22 | PDIA6 | Pdia6 | 298 | TXNIP | Txnip | 574 | IGF2R | Igf2r |
| 23 | SEC61B | Gm10320 | 299 | SCD | Scd2 | 575 | AKT1 | Akt1 |
| 24 | M6PR | M6pr | 300 | SCD | Scd4 | 576 | USP19 | Usp19 |
| 25 | VCAM1 | Vcam1 | 301 | SRPRA | Srpr | 577 | JPH3 | Jph3 |
| 26 | TRIM13 | Trim13 | 302 | CPT2 | Cpt2 | 578 | JPH1 | Jph1 |
| 27 | BCAP31 | Bcap31 | 303 | HSPA1B | Hspa1b | 579 | SRPRB | Srprb |
| 28 | PPARA | Ppara | 304 | HSPA1B | Hspa1a | 580 | PPIB | Ppib |
| 29 | FOS | Fos | 305 | NPC1 | Npc1 | 581 | ELAVL1 | Elavl1 |
| 30 | SDF2L1 | Sdf2l1 | 306 | TNF | Tnf | 582 | NFKB1 | Nfkb1 |
| 31 | SLC26A2 | Slc26a2 | 307 | GANAB | Ganab | 583 | CISD2 | Cisd2 |
| 32 | SELENOK | Selenok | 308 | ATM | Atm | 584 | SLC8A1 | Slc8a1 |
| 33 | CYCS | Gm10053 | 309 | TNFRSF1A | Tnfrsf1a | 585 | INSIG1 | Insig1 |
| 34 | DNM1L | Dnm1l | 310 | GATA1 | Gata1 | 586 | TUSC3 | Tusc3 |
| 35 | HSPA9 | Hspa9 | 311 | ITPR2 | Itpr2 | 587 | HMOX1 | Hmox1 |
| 36 | SEC23A | Sec23a | 312 | OS9 | Os9 | 588 | PPARG | Pparg |
| 37 | EDN1 | Edn1 | 313 | UBL4A | Ubl4a | 589 | SERPINC1 | Serpinc1 |
| 38 | GJB2 | Gjb2 | 314 | EIF2S1 | Eif2s1 | 590 | BNIP1 | Bnip1 |
| 39 | MYC | Myc | 315 | MCL1 | Mcl1 | 591 | MTTP | Mttp |
| 40 | CYB5R3 | Cyb5r3 | 316 | TRDN | Trdn | 592 | AGTR1 | Agtr1b |
| 41 | RNF139 | Rnf139 | 317 | PRDM10 | Prdm10 | 593 | EDEM2 | Edem2 |
| 42 | CTSB | Ctsb | 318 | CYB5R4 | Cyb5r4 | 594 | MAP2K1 | Map2k1 |
| 43 | TGFB3 | Tgfb3 | 319 | TRPC4 | Trpc4 | 595 | GABARAPL1 | Gabarapl1 |
| 44 | EGR1 | Egr1 | 320 | KNG1 | Kng2 | 596 | INSR | Insr |
| 45 | TMED10 | Tmed10 | 321 | KNG1 | Kng1 | 597 | UBXN8 | Ubxn8 |
| 46 | PRL | Prl | 322 | CD4 | Cd4 | 598 | BGLAP | Bglap3 |
| 47 | PRL | Prl3d1 | 323 | SSR1 | Ssr1 | 599 | BGLAP | Bglap2 |
| 48 | PRL | Prl3d2 | 324 | TRIP11 | Trip11 | 600 | BGLAP | Bglap |
| 49 | PRL | Prl3d3 | 325 | PRKAA2 | Prkaa2 | 601 | NUPR1 | Nupr1 |
| 50 | PRL | Prl3c1 | 326 | VKORC1 | Gm21974 | 602 | BRCA1 | Brca1 |
| 51 | PRL | Prl3b1 | 327 | VKORC1 | Vkorc1 | 603 | ACAN | Acan |
| 52 | PRL | Prl3a1 | 328 | CDKN1A | Cdkn1a | 604 | STIP1 | Stip1 |
| 53 | PRL | Prl6a1 | 329 | HMGCR | Hmgcr | 605 | GBA | Gba |
| 54 | PRL | Prl8a2 | 330 | HYOU1 | Hyou1 | 606 | HERPUD1 | Herpud1 |
| 55 | PRL | Prl2b1 | 331 | VIM | Vim | 607 | JPH4 | Jph4 |
| 56 | PRL | Prl8a6 | 332 | CIB1 | Cib1 | 608 | ITPR1 | Itpr1 |
| 57 | PRL | Prl8a8 | 333 | P4HTM | P4htm | 609 | IL6 | Il6 |
| 58 | PRL | Prl8a9 | 334 | MYH7 | Myh7 | 610 | PPARGC1A | Ppargc1a |
| 59 | PRL | Prl8a1 | 335 | HSP90AB1 | Hsp90ab1 | 611 | NOTCH3 | Notch3 |
| 60 | PRL | Prl7b1 | 336 | SLC2A1 | Slc2a1 | 612 | GPR37 | Gpr37 |
| 61 | PRL | Prl7a1 | 337 | SGK1 | Sgk1 | 613 | PON2 | Pon2 |
| 62 | PRL | Prl7a2 | 338 | EDEM1 | Edem1 | 614 | HSPH1 | Hsph1 |
| 63 | PRL | Prl7d1 | 339 | DPAGT1 | Dpagt1 | 615 | TRIB3 | Trib3 |
| 64 | PRL | Prl7c1 | 340 | SSR3 | Ssr3 | 616 | RER1 | Rer1 |
| 65 | PRL | Prl2a1 | 341 | APOA1 | Apoa1 | 617 | HM13 | H13 |
| 66 | PRL | Prl2c1 | 342 | GJA1 | Gja1 | 618 | APOA4 | Apoa4 |
| 67 | PRL | Prl4a1 | 343 | SYVN1 | Syvn1 | 619 | HSPA4 | Hspa4 |
| 68 | PRL | Prl5a1 | 344 | CYP1A1 | Cyp1a1 | 620 | CFTR | Cftr |
| 69 | ERAP1 | Erap1 | 345 | MAPK3 | Mapk3 | 621 | SHH | Shh |
| 70 | SFTPC | Sftpc | 346 | FKRP | Fkrp | 622 | CASQ2 | Casq2 |
| 71 | ESYT2 | Esyt2 | 347 | COMT | Comt | 623 | CREB3L2 | Creb3l2 |
| 72 | HSD17B10 | Hsd17b10 | 348 | OPA3 | Opa3 | 624 | SELENON | Selenon |
| 73 | HLA-DRA | H2-Ea | 349 | NOS3 | Nos3 | 625 | JUN | Jun |
| 74 | UGGT2 | Uggt2 | 350 | DNAJB12 | Dnajb12 | 626 | NCK1 | Nck1 |
| 75 | ERMP1 | Ermp1 | 351 | SHC1 | Shc1 | 627 | G6PC3 | G6pc3 |
| 76 | RSAD2 | Rsad2 | 352 | UBXN4 | Ubxn4 | 628 | FKBP14 | Fkbp14 |
| 77 | MARCHF6 | Marchf6 | 353 | ALG13 | Alg13 | 629 | CNGA3 | Cnga3 |
| 78 | NR3C2 | Nr3c2 | 354 | TFEB | Tfeb | 630 | BAK1 | Bak1 |
| 79 | KCNE1 | Kcne1 | 355 | SCN2A | Scn2a | 631 | RYR2 | Ryr2 |
| 80 | CD8A | Cd8a | 356 | DDOST | Ddost | 632 | STAT3 | Stat3 |
| 81 | AIFM1 | Aifm1 | 357 | TOMM40 | Tomm40 | 633 | ERO1B | Ero1b |
| 82 | NR3C1 | Nr3c1 | 358 | PML | Pml | 634 | MOGS | Mogs |
| 83 | EDNRA | Ednra | 359 | CALR3 | Calr3 | 635 | KEAP1 | Keap1 |
| 84 | ITIH4 | Itih4 | 360 | SEC22B | Sec22b | 636 | CANT1 | Cant1 |
| 85 | C1R | C1ra | 361 | ITGB1 | Itgb1 | 637 | CTNNB1 | Ctnnb1 |
| 86 | C1R | C1rb | 362 | STX18 | Stx18 | 638 | MGAT5 | Mgat5 |
| 87 | FABP1 | Fabp1 | 363 | APOB | Apob | 639 | CHERP | Cherp |
| 88 | STING1 | Sting1 | 364 | ALB | Alb | 640 | TMED9 | Tmed9 |
| 89 | CUL1 | Cul1 | 365 | CDK5 | Cdk5 | 641 | SQSTM1 | Sqstm1 |
| 90 | TAP1 | Tap1 | 366 | PLOD2 | Plod2 | 642 | ATF4 | Atf4 |
| 91 | LMAN1 | Lman1 | 367 | CRH | Crh | 643 | CREB1 | Creb1 |
| 92 | IAPP | Iapp | 368 | RUNX2 | Runx2 | 644 | USO1 | Uso1 |
| 93 | FAS | Fas | 369 | UBC | Ubc | 645 | CAPN1 | Capn1 |
| 94 | TG | Tg | 370 | GAPDH | Gm10358 | 646 | PSMA5 | Psma5 |
| 95 | UFD1 | Ufd1 | 371 | RBX1 | Rbx1 | 647 | UBE2D1 | Ube2d1 |
| 96 | LRP5 | Lrp5 | 372 | ERGIC3 | Ergic3 | 648 | UBB | Ubb |
| 97 | UBQLN2 | Ubqln2 | 373 | PPP1R15B | Ppp1r15b | 649 | MATN3 | Matn3 |
| 98 | MDM2 | Mdm2 | 374 | MBTPS1 | Mbtps1 | 650 | ADAM10 | Adam10 |
| 99 | GOLPH3 | Golph3 | 375 | CXCR4 | Cxcr4 | 651 | TF | Trf |
| 100 | TERT | Tert | 376 | UGT1A1 | Ugt1a1 | 652 | TRPV4 | Trpv4 |
| 101 | TFG | Tfg | 377 | CAV3 | Cav3 | 653 | PRDX5 | Prdx5 |
| 102 | RPN1 | Rpn1 | 378 | TRPC1 | Trpc1 | 654 | RNFT1 | Rnft1 |
| 103 | CLCN1 | Clcn1 | 379 | PPP1CA | Ppp1ca | 655 | MAPK8IP1 | Mapk8ip1 |
| 104 | COL1A2 | Col1a2 | 380 | PXN | Pxn | 656 | BRSK2 | Brsk2 |
| 105 | CLU | Clu | 381 | PINK1 | Pink1 | 657 | HSD17B12 | Hsd17b12 |
| 106 | GLA | Gla | 382 | CYBA | Cyba | 658 | TXN | Txn1 |
| 107 | CKAP4 | Ckap4 | 383 | MYH6 | Myh6 | 659 | INS | Ins2 |
| 108 | PTEN | Pten | 384 | NPLOC4 | Nploc4 | 660 | RYR3 | Ryr3 |
| 109 | GAPDH | Gm3839 | 385 | PDIA3 | Pdia3 | 661 | OXT | Oxt |
| 110 | UBE2D3 | Ube2d2a | 386 | LPL | Lpl | 662 | UFL1 | Ufl1 |
| 111 | KTN1 | Ktn1 | 387 | VEGFA | Vegfa | 663 | SEC11A | Sec11a |
| 112 | UBAC2 | Ubac2 | 388 | SOD2 | Sod2 | 664 | TTPA | Ttpa |
| 113 | EIF2AK3 | Eif2ak3 | 389 | CD36 | Cd36 | 665 | SCG5 | Scg5 |
| 114 | ARSA | Arsa | 390 | SESN2 | Sesn2 | 666 | BOK | Bok |
| 115 | ACP1 | Acp1 | 391 | STUB1 | Stub1 | 667 | DDRGK1 | Ddrgk1 |
| 116 | IFNG | Ifng | 392 | GOSR1 | Gosr1 | 668 | LGI4 | Lgi4 |
| 117 | PDCD6 | Pdcd6 | 393 | ZDHHC6 | Zdhhc6 | 669 | CASP12 | Casp12 |
| 118 | SEL1L | Sel1l | 394 | NOS1 | Nos1 | 670 | JPH2 | Jph2 |
| 119 | PIK3CG | Pik3cg | 395 | QDPR | Qdpr | 671 | ALOX15 | Alox15 |
| 120 | PDIA4 | Pdia4 | 396 | PLN | Pln | 672 | ACTC1 | Actc1 |
| 121 | TLR2 | Tlr2 | 397 | CACNA1C | Cacna1c | 673 | MMP9 | Mmp9 |
| 122 | HLA-DRB1 | H2-Eb2 | 398 | PRL | Prl2c3 | 674 | COL1A1 | Col1a1 |
| 123 | PIK3R1 | Pik3r1 | 399 | PRL | Prl2c2 | 675 | BAX | Bax |
| 124 | ANXA5 | Anxa5 | 400 | PRL | Prl2c5 | 676 | CCDC47 | Ccdc47 |
| 125 | MIA2 | Mia2 | 401 | SLC37A4 | Slc37a4 | 677 | TP53 | Trp53 |
| 126 | CASP2 | Casp2 | 402 | FLNB | Flnb | 678 | CD40 | Cd40 |
| 127 | HFE | Hfe | 403 | HSD11B2 | Hsd11b2 | 679 | YKT6 | Ykt6 |
| 128 | PRKCD | Prkcd | 404 | YOD1 | Yod1 | 680 | PRNP | Prnp |
| 129 | CALM1 | Calm1 | 405 | SCAP | Scap | 681 | CASQ1 | Casq1 |
| 130 | SEC61A1 | Sec61a1 | 406 | OSBP | Osbp | 682 | NOTCH1 | Notch1 |
| 131 | PRDX4 | Prdx4 | 407 | DNAH8 | Dnah8 | 683 | F2 | F2 |
| 132 | CPQ | Cpq | 408 | ERP27 | Erp27 | 684 | SERPINH1 | Serpinh1 |
| 133 | MBTPS2 | Mbtps2 | 409 | DMPK | Dmpk | 685 | TH | Th |
| 134 | C1S | C1s1 | 410 | ERLEC1 | Erlec1 | 686 | ERN2 | Ern2 |
| 135 | C1S | C1s2 | 411 | PDIA2 | Pdia2 | 687 | THBS1 | Thbs1 |
| 136 | TAP2 | Tap2 | 412 | SAR1A | Sar1a | 688 | TOR1A | Tor1a |
| 137 | PTK2 | Ptk2 | 413 | LIPC | Lipc | 689 | UBE2J1 | Ube2j1 |
| 138 | DNAJC3 | Dnajc3 | 414 | MGST1 | Mgst1 | 690 | ELN | Eln |
| 139 | NHLRC1 | Nhlrc1 | 415 | GBF1 | Gbf1 | 691 | C9orf72 | C9orf72 |
| 140 | JKAMP | Jkamp | 416 | SAR1B | Sar1b | 692 | NLRP1 | Nlrp1a |
| 141 | JKAMP | 4931417E11Rik | 417 | ATF3 | Atf3 | 693 | NLRP1 | Nlrp1b |
| 142 | WNT1 | Wnt1 | 418 | ADAMTSL1 | Adamtsl1 | 694 | GABARAP | Gabarap |
| 143 | ALG1 | Alg1 | 419 | CYP1A2 | Cyp1a2 | 695 | EEF2 | Eef2 |
| 144 | SERPINA1 | Serpina1b | 420 | MAP1LC3A | Map1lc3a | 696 | UBE2G2 | Ube2g2 |
| 145 | SERPINA1 | Serpina1d | 421 | ESR1 | Esr1 | 697 | DUSP19 | Dusp19 |
| 146 | SERPINA1 | Serpina1a | 422 | TPP1 | Tpp1 | 698 | ZFAND2B | Zfand2b |
| 147 | SERPINA1 | Serpina1c | 423 | MAP3K5 | Map3k5 | 699 | DRD5 | Drd5 |
| 148 | SERPINA1 | Serpina1e | 424 | INS | Ins1 | 700 | ERN1 | Ern1 |
| 149 | STT3A | Stt3a | 425 | ICMT | Icmt | 701 | SCN4A | Scn4a |
| 150 | CAMK2G | Camk2g | 426 | SHISA5 | Shisa5 | 702 | UBE2G1 | Ube2g1 |
| 151 | BAG6 | Bag6 | 427 | SERP1 | Serp1 | 703 | SNAP25 | Snap25 |
| 152 | CTSK | Ctsk | 428 | FASLG | Fasl | 704 | BMP2 | Bmp2 |
| 153 | HSPA1L | Hspa1l | 429 | SEC13 | Sec13 | 705 | SEC31A | Sec31a |
| 154 | RCN2 | Rcn2 | 430 | GSR | Gsr | 706 | CYP2E1 | Cyp2e1 |
| 155 | GAPDH | Gapdh | 431 | UBQLN4 | Ubqln4 | 707 | ARSG | Arsg |
| 156 | CASP3 | Casp3 | 432 | KCNH2 | Kcnh2 | 708 | RNF185 | Rnf185 |
| 157 | LMAN2 | Lman2 | 433 | EBP | Ebp | 709 | TMEM33 | Tmem33 |
| 158 | HSPA1A | Hspa1b | 434 | MAPK9 | Mapk9 | 710 | CTSD | Gm49369 |
| 159 | HSPA1A | Hspa1a | 435 | PTH | Pth | 711 | CTSD | Ctsd |
| 160 | HSF1 | Hsf1 | 436 | GSTM1 | Gstm6 | 712 | ALG2 | Alg2 |
| 161 | CALR | Calr | 437 | GSTM1 | Gstm3 | 713 | XBP1 | Xbp1 |
| 162 | FMR1 | Fmr1 | 438 | GSTM1 | Gstm2 | 714 | BCL2L11 | Bcl2l11 |
| 163 | TMEM117 | Tmem117 | 439 | GSTM1 | Gstm1 | 715 | ATF6 | Atf6 |
| 164 | MFN2 | Mfn2 | 440 | DERL3 | Derl3 | 716 | TGM2 | Tgm2 |
| 165 | CAV1 | Cav1 | 441 | SKP1 | Skp1 | 717 | PTGIS | Ptgis |
| 166 | TMX1 | Tmx1 | 442 | CRYAA | Cryaa | 718 | TMED4 | Tmed4 |
| 167 | FOXRED2 | Foxred2 | 443 | PIEZO1 | Piezo1 | 719 | CST3 | Cst3 |
| 168 | DSPP | Dspp | 444 | GFAP | Gfap | 720 | CST3 | Cst10 |
| 169 | NLRP3 | Nlrp3 | 445 | SREBF1 | Srebf1 | 721 | IL1B | Il1b |
| 170 | PKD1 | Pkd1 | 446 | UBE2J2 | Ube2j2 | 722 | ABCC8 | Abcc8 |
| 171 | HIF1A | Hif1a | 447 | EIF2AK2 | Eif2ak2 | 723 | KCNJ11 | Kcnj11 |
| 172 | AGR2 | Agr2 | 448 | APEX1 | Apex1 | 724 | RRBP1 | Rrbp1 |
| 173 | RTN3 | Rtn3 | 449 | UBQLN1 | Ubqln1 | 725 | SGTA | Sgta |
| 174 | NQO1 | Nqo1 | 450 | CREBRF | Crebrf | 726 | PTPN11 | Ptpn11 |
| 175 | SPP1 | Spp1 | 451 | AQP11 | Aqp11 | 727 | COPA | Copa |
| 176 | CYCS | Cycs | 452 | COPB1 | Copb1 | 728 | DPM2 | Dpm2 |
| 177 | FOXO3 | Foxo3 | 453 | TNFSF10 | Tnfsf10 | 729 | ACE | Ace |
| 178 | ATXN3 | Atxn3 | 454 | MMP2 | Mmp2 | 730 | ABL1 | Abl1 |
| 179 | IGF2BP2 | Igf2bp2 | 455 | NNT | Nnt | 731 | BSG | Bsg |
| 180 | TGFB1 | Tgfb1 | 456 | MYOC | Myoc | 732 | EGFR | Egfr |
| 181 | MTOR | Mtor | 457 | SELENOF | Selenof | 733 | RECK | Reck |
| 182 | IER3IP1 | Gm50364 | 458 | MAPKAPK2 | Mapkapk2 | 734 | DES | Des |
| 183 | FAF2 | Faf2 | 459 | KDR | Kdr | 735 | TRAF2 | Traf2 |
| 184 | AIF1 | Aif1 | 460 | HEXA | Hexa | 736 | NFE2L2 | Nfe2l2 |
| 185 | VCL | Vcl | 461 | CLN3 | Cln3 | 737 | CAT | Cat |
| 186 | HSP90B1 | Hsp90b1 | 462 | ERO1A | Ero1a | 738 | RAB2A | Rab2a |
| 187 | DSP | Dsp | 463 | HSD11B1 | Hsd11b1 | 739 | STAU1 | Stau1 |
| 188 | APAF1 | Apaf1 | 464 | EIF4E | Eif4e | 740 | DNAJB2 | Dnajb2 |
| 189 | PSMD2 | Psmd2 | 465 | LEP | Lep | 741 | SEC16A | Sec16a |
| 190 | TARDBP | Tardbp | 466 | PRKCSH | Prkcsh | 742 | TOR1B | Tor1b |
| 191 | PARK7 | Park7 | 467 | SCN5A | Scn5a | 743 | PTGS1 | Ptgs1 |
| 192 | PMM2 | Pmm2 | 468 | FICD | Ficd | 744 | COG7 | Cog7 |
| 193 | DNAJB1 | Dnajb1 | 469 | CCR6 | Ccr6 | 745 | VCP | Vcp |
| 194 | F9 | F9 | 470 | CASP8 | Casp8 | 746 | ATP2A3 | Atp2a3 |
| 195 | ALG11 | Alg11 | 471 | TMTC3 | Tmtc3 | 747 | TTN | Ttn |
| 196 | PSEN2 | Psen2 | 472 | PRKN | Prkn | 748 | CACNB4 | Cacnb4 |
| 197 | SGPP2 | Sgpp2 | 473 | GET3 | Get3 | 749 | SEC23B | Sec23b |
| 198 | FOXO1 | Foxo1 | 474 | AUP1 | Aup1 | 750 | MAN1B1 | Man1b1 |
| 199 | ATP2A1 | Atp2a1 | 475 | LMNA | Lmna | 751 | CASP9 | Casp9 |
| 200 | RAB10 | Rab10 | 476 | TAPBP | Tapbp | 752 | CSTB | Cstb |
| 201 | MATN1 | Matn1 | 477 | NOS2 | Nos2 | 753 | ELANE | Elane |
| 202 | G6PD | G6pdx | 478 | PRDX1 | Prdx1 | 754 | MAP2 | Map2 |
| 203 | ALG3 | Alg3 | 479 | TYR | Tyr | 755 | DAB2IP | Dab2ip |
| 204 | TMEM43 | Tmem43 | 480 | FKBP5 | Fkbp5 | 756 | TMCO1 | Tmco1 |
| 205 | DNAJB9 | Dnajb9 | 481 | SNCA | Snca | 757 | STX17 | Stx17 |
| 206 | PSEN1 | Psen1 | 482 | EPM2A | Epm2a | 758 | NCK2 | Nck2 |
| 207 | STARD3 | Stard3 | 483 | BECN1 | Becn1 | 759 | STIM1 | Stim1 |
| 208 | BSCL2 | Bscl2 | 484 | SPAST | Spast | 760 | GUSB | Gusb |
| 209 | VWF | Vwf | 485 | MAP2K7 | Map2k7 | 761 | VAPB | Vapb |
| 210 | NPPB | Nppb | 486 | MAP2K7 | Gm49320 | 762 | TMED2 | Tmed2 |
| 211 | INPP5K | Inpp5k | 487 | FURIN | Furin | 763 | NSFL1C | Nsfl1c |
| 212 | HP | Hp | 488 | LRRK2 | Lrrk2 | 764 | CACNA1S | Cacna1s |
| 213 | ANK2 | Ank2 | 489 | RPSA | Rpsa | 765 | SOAT1 | Soat1 |
| 214 | FGFR3 | Fgfr3 | 490 | CERT1 | Cert1 | 766 | SIGMAR1 | Sigmar1 |
| 215 | APP | App | 491 | ICAM1 | Icam1 | 767 | SOX9 | Sox9 |
| 216 | ORMDL3 | Ormdl3 | 492 | ESYT1 | Esyt1 | 768 | EPO | Epo |
| 217 | TMBIM6 | Tmbim6 | 493 | CAPN2 | Capn2 | 769 | CREB3L3 | Creb3l3 |
| 218 | SERPINA7 | Serpina7 | 494 | TFRC | Tfrc | 770 | MAPT | Mapt |
| 219 | VAPA | Vapa | 495 | NOX4 | Nox4 | 771 | EIF2AK4 | Eif2ak4 |
| 220 | TLR4 | Tlr4 | 496 | SSR2 | Ssr2 | 772 | CRP | Crp |
| 221 | ORAI1 | Orai1 | 497 | TRAPPC2 | Trappc2 | 773 | BCL2 | Bcl2 |
| 222 | DHCR24 | Dhcr24 | 498 | MAPK14 | Mapk14 | 774 | SEC61G | Sec61g |
| 223 | COL2A1 | Col2a1 | 499 | HTRA2 | Htra2 | 775 | TMEM259 | Tmem259 |
| 224 | SERPINA3 | Serpina3a | 500 | STBD1 | Stbd1 | 776 | CREB3L1 | Creb3l1 |
| 225 | SERPINA3 | Serpina3b | 501 | DCN | Dcn | 777 | DNAJC10 | Dnajc10 |
| 226 | SERPINA3 | Serpina3c | 502 | CCDC88B | Ccdc88b | 778 | ERP44 | Erp44 |
| 227 | SERPINA3 | Serpina3f | 503 | OSBPL8 | Osbpl8 | 779 | PNKD | Pnkd |
| 228 | SERPINA3 | Serpina3g | 504 | WFS1 | Wfs1 | 780 | NFE2L1 | Nfe2l1 |
| 229 | SERPINA3 | Serpina3i | 505 | CP | Cp | 781 | GET4 | Get4 |
| 230 | SERPINA3 | Serpina3j | 506 | MAN1A1 | Man1a | 782 | KCNQ1 | Kcnq1 |
| 231 | SERPINA3 | Serpina3k | 507 | BACE1 | Bace1 | 783 | GPER1 | Gper1 |
| 232 | SERPINA3 | Serpina3m | 508 | FUS | Fus | 784 | PRKCA | Prkca |
| 233 | SERPINA3 | Serpina3n | 509 | CALHM1 | Calhm1 | 785 | KDELR1 | Kdelr1 |
| 234 | RB1 | Rb1 | 510 | DYSF | Dysf | 786 | LDLR | Ldlr |
| 235 | SGPP1 | Sgpp1 | 511 | IL10 | Il10 | 787 | LNPK | Lnpk |
| 236 | EEF1A1 | Eef1a1 | 512 | HTT | Htt | 788 | POR | Por |
| 237 | SIRT1 | Sirt1 | 513 | GORASP2 | Gorasp2 | 789 | PTPN1 | Ptpn1 |
| 238 | EDEM3 | Edem3 | 514 | ZC3H12A | Zc3h12a | 790 | DERL2 | Derl2 |
| 239 | EGF | Egf | 515 | CANX | Canx | 791 | DHCR7 | Dhcr7 |
| 240 | TNFSF11 | Tnfsf11 | 516 | GSTP1 | Gstp1 | 792 | G3BP1 | G3bp1 |
| 241 | ATF6B | Atf6b | 517 | GSTP1 | Gstp2 | 793 | BDNF | Bdnf |
| 242 | DNAJB11 | Dnajb11 | 518 | FKBP10 | Fkbp10 | 794 | MPO | Mpo |
| 243 | TEX264 | Tex264 | 519 | MAPK8 | Mapk8 | 795 | SVIP | Svip |
| 244 | F8 | F8 | 520 | COL9A1 | Col9a1 | 796 | PTGS2 | Ptgs2 |
| 245 | EIF2AK1 | Eif2ak1 | 521 | TRPM4 | Trpm4 | 797 | BBC3 | Bbc3 |
| 246 | DDIT3 | Ddit3 | 522 | GP6 | Gp6 | 798 | AVP | Avp |
| 247 | TMX3 | Tmx3 | 523 | ITPR3 | Itpr3 | 799 | CEBPB | Cebpb |
| 248 | SEC24C | Sec24c | 524 | XDH | Xdh | 800 | KPNB1 | Kpnb1 |
| 249 | RNF5 | Rnf5 | 525 | AMFR | Amfr | 801 | UBE2K | Ube2k |
| 250 | CYP21A2 | Cyp21a1 | 526 | KRT14 | Krt14 | 802 | RAB6A | Rab6a |
| 251 | HSPA2 | Hspa2 | 527 | GPX1 | Gpx1 | 803 | KDELR2 | Kdelr2 |
| 252 | ENO2 | Eno2 | 528 | RYR1 | Ryr1 | 804 | ATXN2 | Atxn2 |
| 253 | RTN1 | Rtn1 | 529 | SLC6A4 | Slc6a4 | 805 | DPM1 | Gm20716 |
| 254 | ATP2A2 | Atp2a2 | 530 | CDIPT | Cdipt | 806 | DPM1 | Dpm1 |
| 255 | OSBPL3 | Osbpl3 | 531 | PON1 | Pon1 | 807 | HSPB1 | Hspb1 |
| 256 | IGF1 | Igf1 | 532 | UBA52 | Uba52 | 808 | RCN1 | Rcn1 |
| 257 | TMED7 | Tmed7 | 533 | TMEM208 | Tmem208 | 809 | RPN2 | Rpn2 |
| 258 | CCND1 | Ccnd1 | 534 | COMP | Comp | 810 | SRC | Src |
| 259 | CDK1 | Cdk1 | 535 | IL7 | Il7 | 811 | PPP1R15A | Ppp1r15a |
| 260 | SEC24B | Sec24b | 536 | NGF | Ngf | 812 | RAB1A | Rab1a |
| 261 | RHBDD1 | Rhbdd1 | 537 | UGGT1 | Uggt1 | 813 | TNFRSF10B | Tnfrsf26 |
| 262 | PCSK9 | Pcsk9 | 538 | SEC23IP | Sec23ip | 814 | TNFRSF10B | Tnfrsf22 |
| 263 | GCG | Gcg | 539 | SCAMP5 | Scamp5 | 815 | TNFRSF10B | Tnfrsf23 |
| 264 | MANF | Manf | 540 | TMCC1 | Tmcc1 | 816 | SEC63 | Sec63 |
| 265 | PKD2 | Pkd2 | 541 | SRP68 | Srp68 | 817 | RAC1 | Rac1 |
| 266 | SEC31B | Sec31b | 542 | CDKN3 | Cdkn3 | 818 | BAG1 | Bag1 |
| 267 | NPY | Npy | 543 | TCF7L2 | Tcf7l2 | 819 | ECPAS | Ecpas |
| 268 | H6PD | H6pd | 544 | RHOA | Rhoa | 820 | HSPA5 | Hspa5 |
| 269 | TXNDC5 | Txndc5 | 545 | U2AF1 | U2af1 | 821 | PIGN | Pign |
| 270 | AGR3 | Agr3 | 546 | SELENOS | Selenos | 822 | ERP29 | Erp29 |
| 271 | AGER | Ager | 547 | AFP | Afp | 823 | SERPINE1 | Serpine1 |
| 272 | ERGIC2 | Ergic2 | 548 | SP1 | Sp1 | 824 | CREB3 | Creb3 |
| 273 | AGTR1 | Agtr1a | 549 | TIA1 | Tia1 | 825 | FN1 | Fn1 |
| 274 | GPT | Gpt | 550 | NGLY1 | Ngly1 | 826 | ARL6IP1 | Arl6ip1 |
| 275 | UFM1 | Ufm1 | 551 | HSPA8 | Hspa8 | 827 | ASPH | Asph |
| 276 | SCFD1 | Scfd1 | 552 | BCL2L1 | Bcl2l1 | 828 | TMEM214 | Tmem214 |

**Supplementary Table 3.** Primers for qPCR used in the current study

| Primer | Sequence | |
| --- | --- | --- |
| Cdkn1a F | TACGGCAACACTGGGTAACC | |
| Cdkn1a R | GACCATCTGGGGTGGTGTAAG | |
| Atf3 F | AGGACGATGACAGGAAAGTTCG |  |
| Atf3 R | TGCACAAAGTTCATAGGACACAG | |
| Fkbp5 F | GATGAGGGCACCAGTAACAATG |  |
| Fkbp5 R | CAACATCCCTTTGTAGTGGACAT |  |
| Gabarapl1 F | GGACCACCCCTTCGAGTATC | |
| Gabarapl1 R | CCTCTTATCCAGATCAGGGACC |  |
| Serpina3n F | CAACCAGAGACCCTGAGGAAGT | |
| Serpina3n R | AGGACATCCTCCAGGCTGTAGT |  |
| Nos2 F | GAGACAGGGAAGTCTGAAGCAC |  |
| Nos2 R | CCAGCAGTAGTTGCTCCTCTTC |  |
| Tfrc F | GAAGTCCAGTGTGGGAACAGGT |  |
| Tfrc R | CAACCACTCAGTGGCACCAACA |  |
| Nox4 F | CGGGATTTGCTACTGCCTCCAT |  |
| Nox4 R | GTGACTCCTCAAATGGGCTTCC |  |
| Herpud1 F | CCTCCAAAATGCCAGAAACCAGC |  |
| Herpud1 R | GCCGTAAACCATCACTTGAGGAG |  |
| Casq1 F | TGGTGGACTCAGAGAAGGATGC |  |
| Casq1 R | AACTCCACCAGAGTGTCTGCAG |  |
| Ppp1r15a F | GGCGGCTCAGATTGTTCAAAGC |  |
| Ppp1r15a R | CCAGACAGCAAGGAAATGGACTG |  |
| Hp F | ACGGCTATGTGGAGCACTTGGT |  |
| Hp R | GTTTCTCTCCAGCGACTGTGTTC |  |
| Fos F | GGGAATGGTGAAGACCGTGTCA |  |
| Fos R | GCAGCCATCTTATTCCGTTCCC |  |
| internal reference GAPDH F | TGGTGAAGGTCGGTGTGAAC |  |
| internal reference GAPDH R | GCTCCTGGAAGATGGTGATGG |  |

**Supplementary Table 4.** The expression levels of DE-ERSRGs in the Case vs Control group
