# Supplementary material for: Transcriptome-wide analysis reveals the molecular mechanisms of cannabinoid type II receptor agonists in cardiac injury induced by chronic psychological stress
Source: Front Genet. 2023 Jan 10;13:1095428. doi: 10.3389/fgene.2022.1095428 (PMC9871316; doi:10.3389/fgene.2022.1095428)
Supplement: Supplementary file 1 [file DataSheet1.zip › Supplementary Table 3 RT-qPCR primers.docx]

**Supplementary** **Table 2: Primers for qPCR used in the current study**

| Primer | Sequence | |
| --- | --- | --- |
| Cdkn1a F | TACGGCAACACTGGGTAACC | |
| Cdkn1a R | GACCATCTGGGGTGGTGTAAG | |
| Atf3 F | AGGACGATGACAGGAAAGTTCG |  |
| Atf3 R | TGCACAAAGTTCATAGGACACAG | |
| Fkbp5 F | GATGAGGGCACCAGTAACAATG |  |
| Fkbp5 R | CAACATCCCTTTGTAGTGGACAT |  |
| Gabarapl1 F | GGACCACCCCTTCGAGTATC | |
| Gabarapl1 R | CCTCTTATCCAGATCAGGGACC |  |
| Serpina3n F | CAACCAGAGACCCTGAGGAAGT | |
| Serpina3n R | AGGACATCCTCCAGGCTGTAGT |  |
| Nos2 F | GAGACAGGGAAGTCTGAAGCAC |  |
| Nos2 R | CCAGCAGTAGTTGCTCCTCTTC |  |
| Tfrc F | GAAGTCCAGTGTGGGAACAGGT |  |
| Tfrc R | CAACCACTCAGTGGCACCAACA |  |
| Nox4 F | CGGGATTTGCTACTGCCTCCAT |  |
| Nox4 R | GTGACTCCTCAAATGGGCTTCC |  |
| Herpud1 F | CCTCCAAAATGCCAGAAACCAGC |  |
| Herpud1 R | GCCGTAAACCATCACTTGAGGAG |  |
| Casq1 F | TGGTGGACTCAGAGAAGGATGC |  |
| Casq1 R | AACTCCACCAGAGTGTCTGCAG |  |
| Ppp1r15a F | GGCGGCTCAGATTGTTCAAAGC |  |
| Ppp1r15a R | CCAGACAGCAAGGAAATGGACTG |  |
| Hp F | ACGGCTATGTGGAGCACTTGGT |  |
| Hp R | GTTTCTCTCCAGCGACTGTGTTC |  |
| Fos F | GGGAATGGTGAAGACCGTGTCA |  |
| Fos R | GCAGCCATCTTATTCCGTTCCC |  |
| internal reference GAPDH F | TGGTGAAGGTCGGTGTGAAC |  |
| internal reference GAPDH R | GCTCCTGGAAGATGGTGATGG |  |
