# Supplementary figures and images for: Transcriptome-wide analysis reveals the molecular mechanisms of cannabinoid type II receptor agonists in cardiac injury induced by chronic psychological stress
Source: Front Genet. 2023 Jan 10;13:1095428. doi: 10.3389/fgene.2022.1095428 (PMC9871316; doi:10.3389/fgene.2022.1095428)

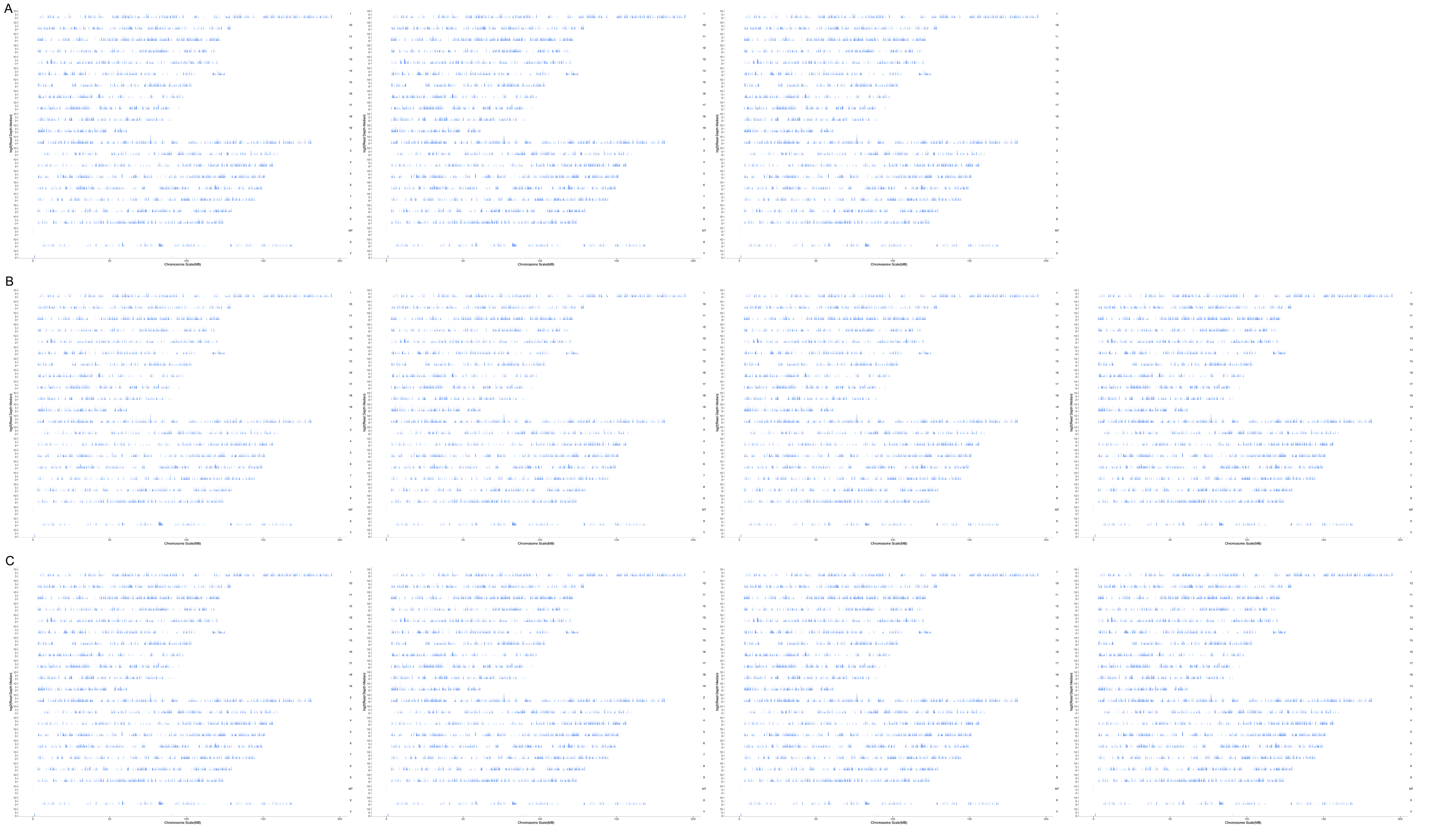

Supplement: Supplementary file 1 [file DataSheet1.zip › Supplementary Figure 1.tif]

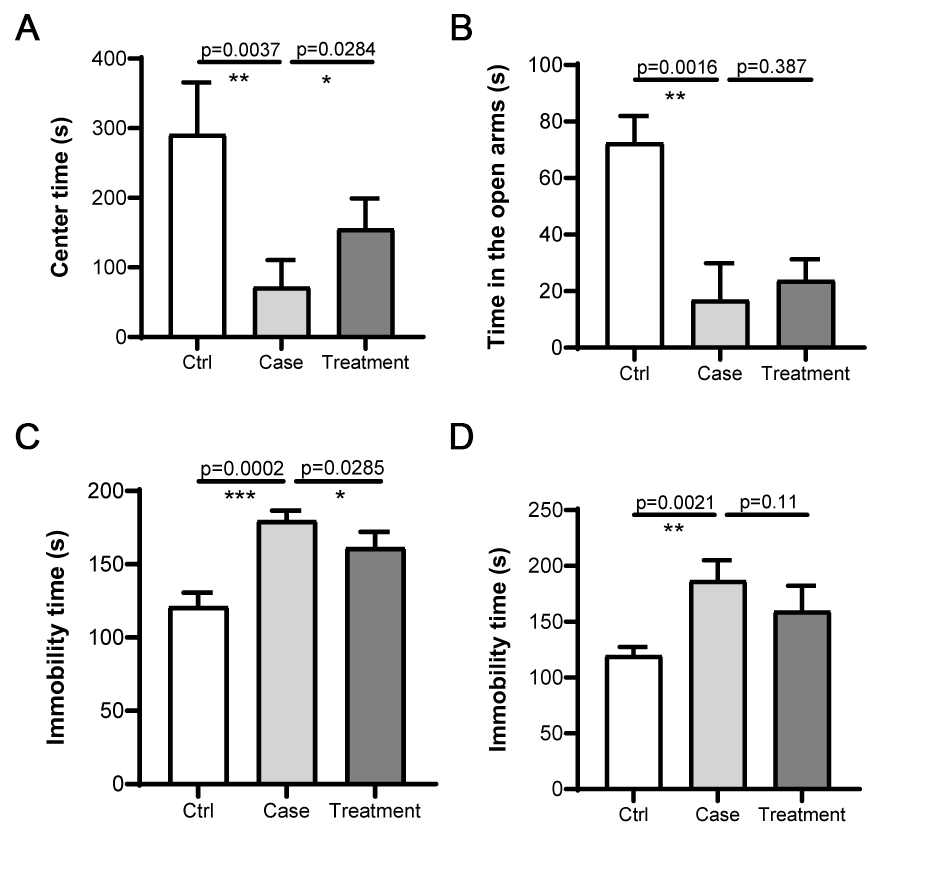

Supplement: Supplementary file 1 [file DataSheet1.zip › Supplementary Figure 2.tif]

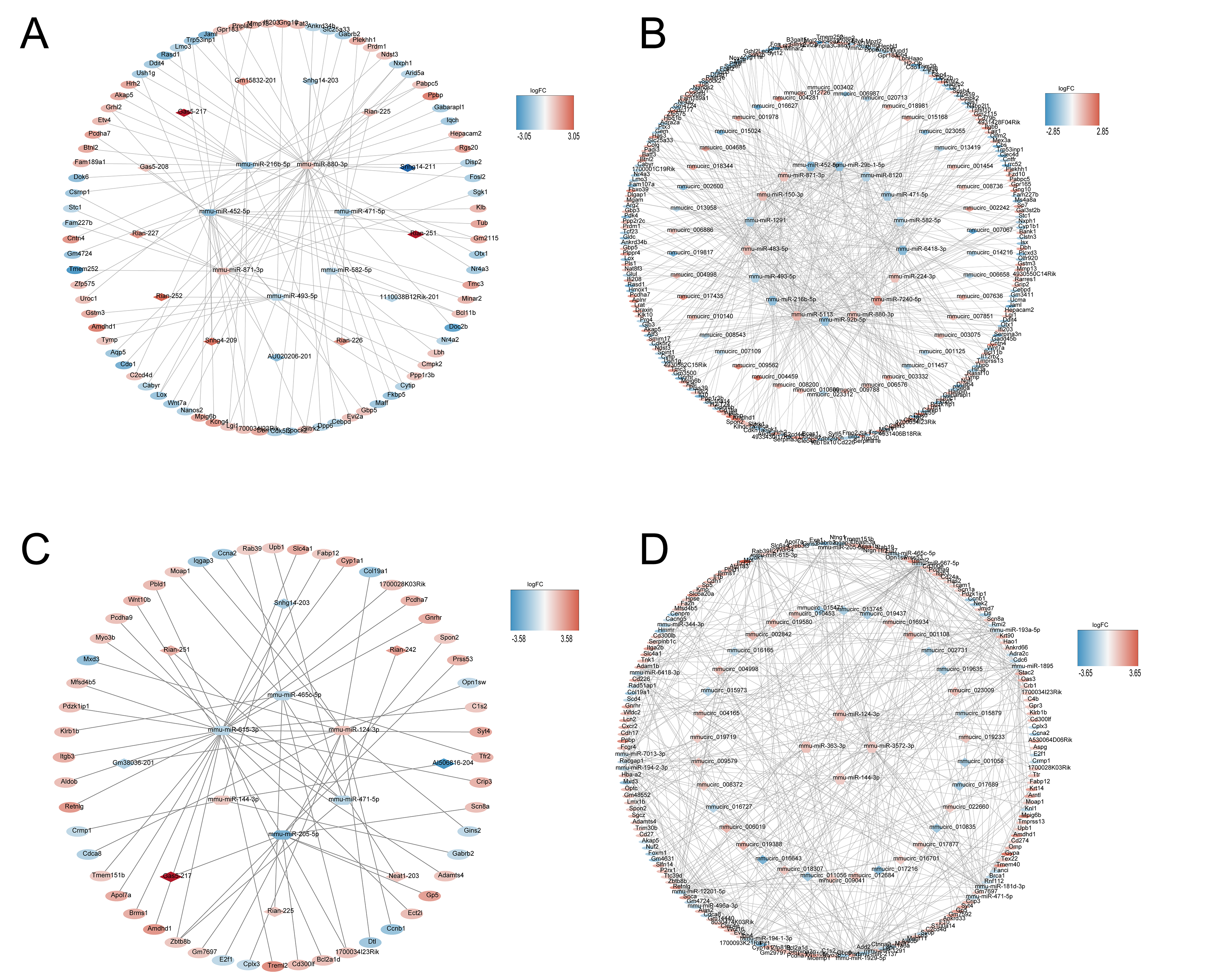

Supplement: Supplementary file 1 [file DataSheet1.zip › Supplementary Figure 4.tif]

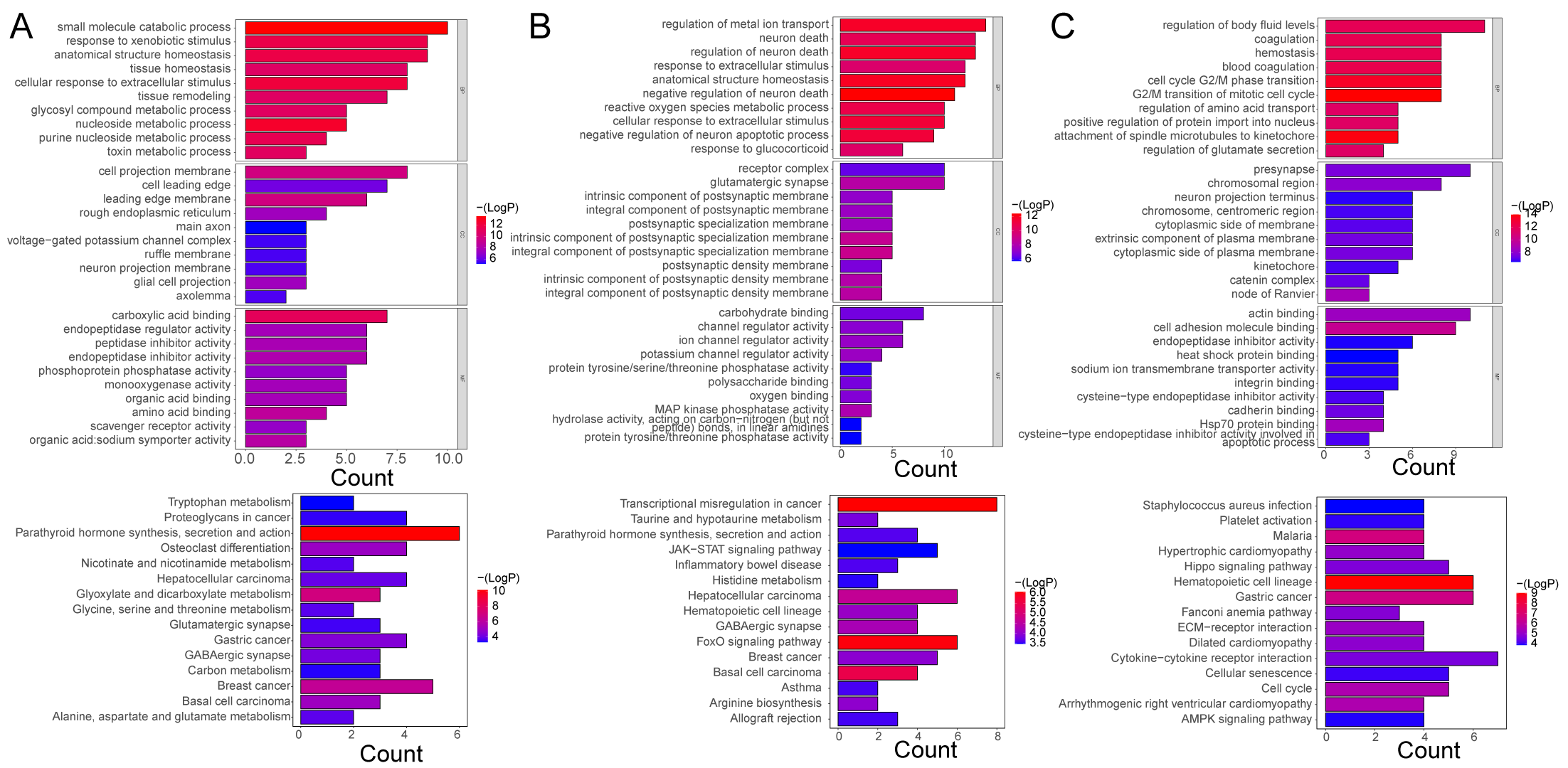

Supplement: Supplementary file 1 [file DataSheet1.zip › Supplementary Figure 5.tif]

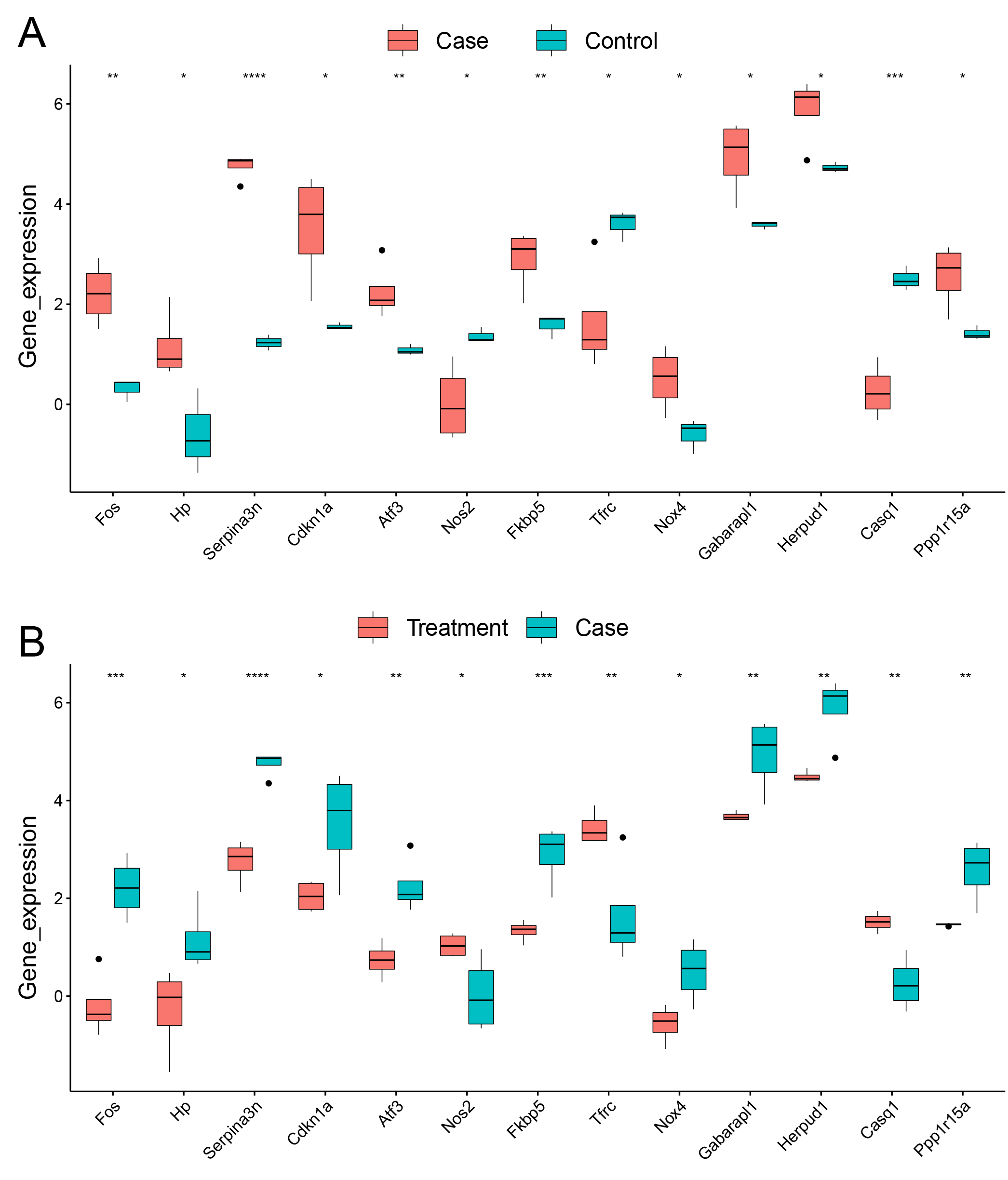

Supplement: Supplementary file 1 [file DataSheet1.zip › Supplementary Figure 6.tif]

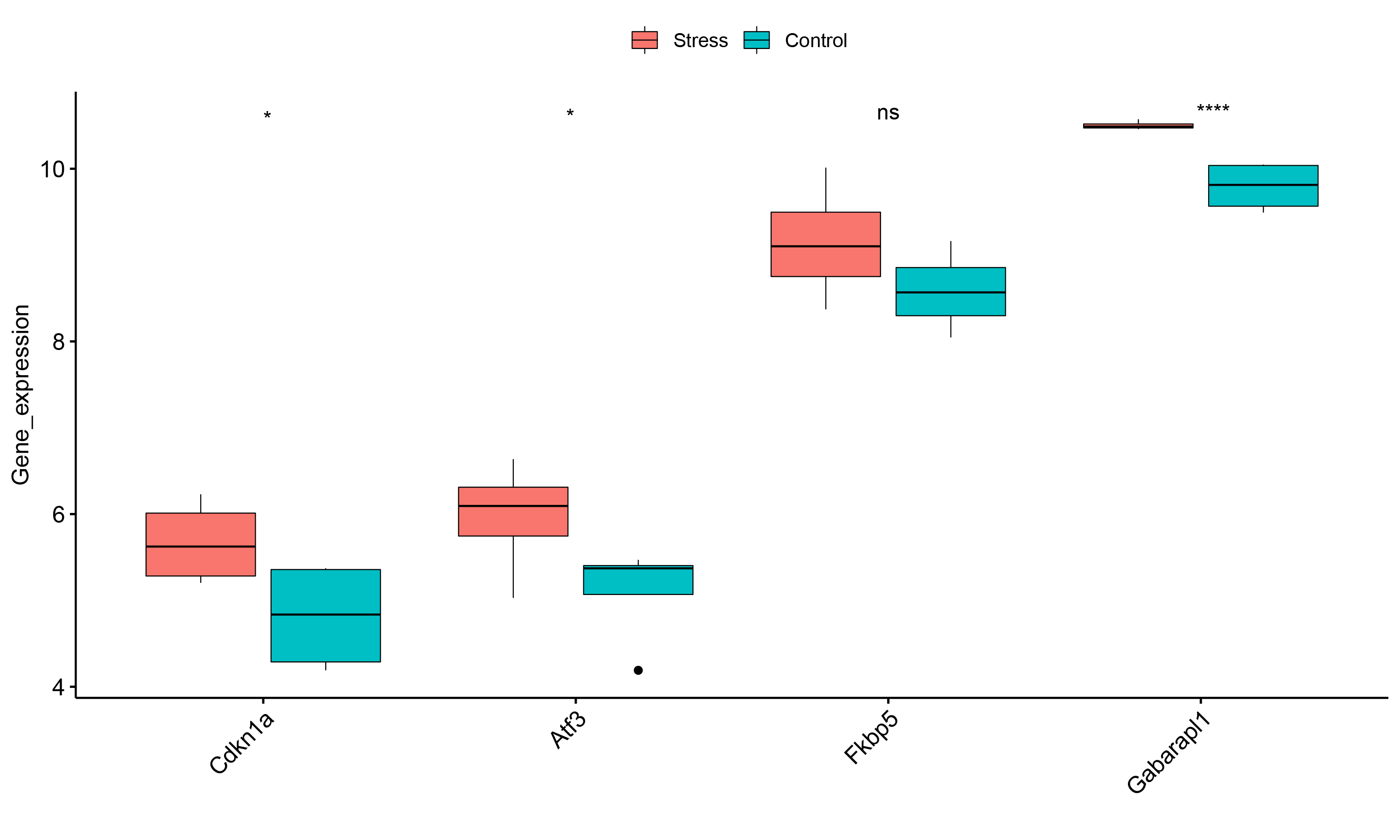

Supplement: Supplementary file 1 [file DataSheet1.zip › Supplementary Figure 7.tif]
